# Uncovering the Uncultivated Majority in Antarctic Soils: Toward a Synergistic Approach

**DOI:** 10.3389/fmicb.2019.00242

**Published:** 2019-02-15

**Authors:** Sam Lambrechts, Anne Willems, Guillaume Tahon

**Affiliations:** Laboratory of Microbiology, Department of Biochemistry and Microbiology, Ghent University, Ghent, Belgium

**Keywords:** Antarctica, uncultivated majority, cultivation, terrestrial, cultivation-independent, metagenomics, candidate phyla, microbial dark matter

## Abstract

Although Antarctica was once believed to be a sterile environment, it is now clear that the microbial communities inhabiting the Antarctic continent are surprisingly diverse. Until the beginning of the new millennium, little was known about the most abundant inhabitants of the continent: prokaryotes. From then on, however, the rising use of deep sequencing techniques has led to a better understanding of the Antarctic prokaryote diversity and provided insights in the composition of prokaryotic communities in different Antarctic environments. Although these cultivation-independent approaches can produce millions of sequences, linking these data to organisms is hindered by several problems. The largest difficulty is the lack of biological information on large parts of the microbial tree of life, arising from the fact that most microbial diversity on Earth has never been characterized in laboratory cultures. These unknown prokaryotes, also known as microbial dark matter, have been dominantly detected in all major environments on our planet. Laboratory cultures provide access to the complete genome and the means to experimentally verify genomic predictions and metabolic functions and to provide evidence of horizontal gene transfer. Without such well-documented reference data, microbial dark matter will remain a major blind spot in deep sequencing studies. Here, we review our current understanding of prokaryotic communities in Antarctic ice-free soils based on cultivation-dependent and cultivation-independent approaches. We discuss advantages and disadvantages of both approaches and how these strategies may be combined synergistically to strengthen each other and allow a more profound understanding of prokaryotic life on the frozen continent.

## Introduction

Although the microbial tree of life has greatly expanded over the last decades, from Woese’s 12 phyla to more recent estimates of at least 310 phyla (Brown et al., 2015; Hug et al., 2016; Parks et al., 2017; Zaremba-Niedzwiedzka et al., 2017; Castelle et al., 2018), much is still to be discovered as estimates suggest that between 10^11^-10^12^ microbial species inhabit Earth (Locey and Lennon, 2016). Documented sequence information comprises some 10^5^ species of which only a minority (∼12,700) have successfully been cultured (Marx, 2017)^1^. For many environments analyses indicate that between 0.1 and 1% of the currently known microorganisms have been cultivated (Vartoukian et al., 2010; Solden et al., 2016). The bulk of the microbial diversity thus still remains uncultivated. These uncultured, and in most cases also unknown prokaryotes are often referred to as microbial dark matter because not only do they likely account for a large portion of our planet’s biomass and are numerically dominant in most of its environments, but also their metabolic properties and ecological significance remain unknown (Solden et al., 2016; Lloyd et al., 2018). Thus, although microorganisms are without doubt the most abundant and diverse cellular life forms on Earth, our knowledge about them is biased by the very minute number that have been cultivated in the lab. In today’s genomic era, cultivation is no longer a prerequisite for obtaining information on microbial dark matter. Nevertheless, without laboratory cultures, their predicted growth characteristics, metabolism and physiology cannot be unequivocally determined, leaving many unanswered questions regarding their role and significance in the environment.

The microbial communities in various extreme environments worldwide have received increasing research interest over the last decades. They frequently harbor a wide diversity of prokaryotes (Tindall, 2004) able to cope with extreme conditions and knowledge about their biology may thus be very important for novel industrial, medical and commercial applications (Perfumo et al., 2018). Also, Antarctic microbial communities have been increasingly studied (e.g., Lamilla et al., 2017; Lee et al., 2018; Yarzábal et al., 2018). Despite being nearly completely covered by a thick layer of ice, Antarctica, the largest desert on Earth, harbors a wide range of aquatic and terrestrial environments. Many of these are considered as the last pristine places on Earth and have been exposed to relatively limited anthropogenic intervention (Teixeira et al., 2010). However, in recent years, the increase in research activity and tourism has resulted in some direct, occasionally intense effects on Antarctic microbial communities through contamination (e.g., oil spills, alien species introductions), eutrophication and transportation of microorganisms between different sites (Cowan et al., 2011).

A small fraction of the total surface area of the continent consists of permanently ice-free regions (Peck et al., 2005). To date, these exposed Antarctic soils have received relatively little research attention, resulting in a rather limited knowledge on their microbial communities. However, their study can lead to improved understanding of how life thrives under these environmental conditions (Dedysh, 2011; De Maayer et al., 2014). The purpose of this review is to provide an update on the prokaryotic diversity of continental Antarctic soils in ice-free regions with a particular focus on uncultivated groups, the challenges of cultivation, and how a synergistic combination of different methodologies can increase the cultivation rate of this microbial dark matter, leading to better understanding of the role and potential of these microorganisms.

## The Antarctic Continent

Ever since its discovery in 1820, Antarctica, with a surface area of ∼13.6–14 million km^2^ about 1.4 times the size of Europe, proved to be a great challenge for explorers (Kennedy, 1995; Chown and Convey, 2007). While throughout most of its history the continent’s climate was temperate to sub-tropical, approximately 33.7 million years ago, a combination of gradually declining atmospheric CO_2_ partial pressure and tectonic reorganizations led to the onset of a large-scale glaciation of Antarctica (Convey et al., 2008; Elsworth et al., 2017). Furthermore, due to its geographical location and its isolation (Antarctica is nearly completely enclosed within the Antarctic Polar Circle, Supplementary Figure S1), Antarctica is now an ice-covered land mass which has been isolated from the rest of the world for over 10 million years (Francis, 1988; Zachos et al., 2001; Baker et al., 2003; Bargagli, 2005).

Antarctica can be divided into four major geographical regions: West and East Antarctica, the Antarctic Peninsula and the Sub-Antarctic region (Supplementary Figure S1). West and East Antarctica are by far the largest units, separated from each other by one of the longest (∼3500 km) mountain ranges on our planet, the Transantarctic Mountains (Convey et al., 2009; Turner et al., 2009). West Antarctica is the ‘lower’ part of the continent (on average 850 m high) and is covered by the West-Antarctic ice sheet (∼1.97 M km^2^). East Antarctica, on the other hand, comprises about two-thirds of the total area of the continent and is covered by the much thicker East-Antarctic ice sheet with an average altitude of 2300 m (Bargagli, 2005, 2008; Convey et al., 2009). The Antarctic Peninsula, sometimes considered part of West Antarctica and covered by the remains of what once was the Antarctic Peninsula ice sheet, is the only part of the continent that extends a significant way northward from the main ice sheet, its tip close the 63° S circle of latitude (Supplementary Figure S1) (Convey et al., 2009; Davies et al., 2012). With an average width of 70 km and a mean height of 1500 m, this mountainous region is a bedrock archipelago that acts as a barrier between eastern and westerns atmospheric and oceanic circulations (Bargagli, 2005; Convey et al., 2009). Finally, the Sub-Antarctic region (45–55° S) encompasses a ring of oceanic islands characterized by milder environmental conditions compared to the more Southern regions (Chong et al., 2015).

Antarctica and its inhabitants are constantly exposed to extreme climatic conditions which are characterized by limited organic nutrients, low humidity, frequent freeze-thaw and wet-dry cycles, low temperatures, fluctuating UV radiation and strong desiccating winds (Wynn-Williams, 1990; Cowan and Tow, 2004). Low temperatures are predominantly caused by the permanent ice cover creating an albedo effect (i.e., reflecting solar radiation), the average altitude of over 2 km, and long periods of (nearly) complete darkness (i.e., the austral winter) combined with an aphelion position (Bargagli, 2005, 2008). The record of lowest temperature (-98.6°C) was recorded on Antarctica in 2018 (Scambos et al., 2018). However, yearly average temperatures – the Antarctic Peninsula not included – usually range from -25 to -70°C in winter and -4 to -30°C during the summer, the warmest temperatures appearing in the coastal areas (Bargagli, 2005). Average annual temperatures on the Peninsula are, due to its lower latitude, much higher than elsewhere on the continent, although clear differences can be observed between its west and east coast. While the west coast of the Peninsula is characterized by a mild maritime climate with average annual temperatures of approximately -1.8°C, temperatures at the east coast are about 7°C lower as a result of southerly winds and a greater extent of sea ice (Bargagli, 2005).

As a consequence of the below-zero temperatures and katabatic wind effects, most precipitation sublimates before it reaches the surface (Nylen et al., 2004). The remaining precipitation comes in the form of snow, most of which falls from March to May, although significant regional variation across the continent exists (Marshall, 2009; Cary et al., 2010). Throughout the continent, the amount of precipitation is, however, extremely low, with an average of 175 mm water equivalent each year (Turner et al., 2009; Palerme et al., 2014; Bockheim, 2015a). Antarctica is therefore the largest, but also coldest desert on Earth, since desert climates are characterized by annual precipitation rates of less than 250 mm water equivalent (Nylen et al., 2004).

Although the continent is nearly completely covered by a thick layer of ice, permanently ice-free regions can be found across Antarctica. They are, however, extremely scarce, comprising only 0.32–0.4% of the total surface area and consist mostly of loose, sandy material devoid of plants and of an organic layer (Supplementary Figure S1) (Convey et al., 2008). For a long time there was no consensus on their definition as soil (Jensen, 1916). However, the definition of soil as stated by The Soil Science Society of America (i.e., soil is the layer(s) of generally loose mineral and/or organic material that are affected by physical, chemical, and/or biological processes at or near the planetary surface and usually hold liquids, gasses, and biota and support plants) broadly encompasses the diverse range of Antarctic soil habitats, despite the complete absence of higher plants in all areas other than the Peninsula (Cowan et al., 2014; van Es, 2017). Furthermore, even the most extreme and apparently depauperate ice-free Antarctic areas can be considered as soil habitats, given that they all contain detectable – although sometimes very low – levels of organic carbon and microbial populations (Cowan et al., 2014). Although many different types of Antarctic soils exist, in general, they comprise a surface pavement (i.e., a layer of gravel, stones or boulders formed largely by weathering and the removal of fine materials mainly by wind action) and a seasonally thawed active layer over permafrost. The active layer, often loose and unconsolidated, mostly ranges from only a couple of centimeters up to one meter deep within the soil profile (Bockheim, 2015b).

Antarctic ice-free soils encompass a large range of terrestrial habitats differing in geochemical soil characteristics, physical habitat characteristics and the presence of macroscopic life forms. Abiotic factors known to structure microbial communities in Antarctic soils include nutrient and ion concentrations, water availability, pH, microclimatic conditions and geochemistry (e.g., Cary et al., 2010; Tytgat et al., 2016).

Nutrient-rich soils are often found near the coastal areas where birds are considered a major factor influencing soil organic carbon and nutrient levels. The effects of high levels of carbon (>4%), nitrogen (>2%), and phosphorus (>1%) in these ornithogenic soils are, however, not restricted to areas with direct seabird manure inputs (Aislabie et al., 2009; Bockheim, 2015a). Bird trampling and guano create unfavorable chemical conditions (e.g., low pH, high conductivity) which result in exposed rookery surface soils susceptible to wind and water erosion that can transport nutrients over a wider region (Bockheim, 2015a; Otero et al., 2018).

The majority of Antarctic soils are extremely poor in water (<2%) and nutrients (Kennedy, 1993; Cary et al., 2010; Mergelov et al., 2015; Zazovskaya et al., 2015) as a result of centuries, or even millennia of extreme environmental conditions. Furthermore, the absence of vascular plants and large numbers of primary producers has resulted in soils containing only very low amounts of organic matter (Kennedy, 1993; Cary et al., 2010). Nevertheless, sudden environmental changes may temporarily provide improved conditions for life when, for example, wind erosion deposits allochthonous nutrients, or during summer, meltwater runoff wets soils (Cowan and Tow, 2004; McKnight et al., 2004; Bockheim, 2015a) or very occasionally animal carcasses create a nutrient hotspot supporting the soil’s biota (Nelson et al., 2008; Cary et al., 2010; Tiao et al., 2012).

## Earliest Reports of Prokaryotes on Antarctica

For a long time after the discovery of the continent in 1820, life was thought to be completely absent from Antarctica which was regarded as merely a sterile environment (Chown and Convey, 2007; Convey, 2010). Indeed, we now know that the bulk of Antarctica’s diversity lies in the less visible groups, represented by the microbiota and especially Bacteria (Chown et al., 2015). Currently, Archaea, the second most abundant group, have a considerably lower diversity, although this may be caused by methodological limitations (Bowman, 2018). Although it is unclear when exactly prokaryotes were first found to inhabit Antarctic ecosystems, scientific reports from the early 1900s mention their presence. To our knowledge, the oldest report dates from 1908 and describes the findings of Ekelöf (Ekelöf, 1908). During the Swedish Antarctic expedition (1901–1904), he exposed petri dishes for at least 2 h on Snow Hill Island near Graham Land (64°28′S 57°12′W). On more than half of the exposures, growth could be observed which Ekelöf assumed to originate from microorganisms attached to soil dust that was carried into the dishes by air currents (Ekelöf, 1908).

Around the same time, other researchers also studied Antarctic air. In 1902, during the first German expedition to Antarctica, Gazert indirectly examined the air at Gaussberg (66°48′S 89°11′E) by making cultures from freshly fallen snow. In 1903, during the voyage of the S.Y. Scotia, Pirie examined the bacteriological diversity present in air on the beach at the head of Scotia Bay (60°46′S 44°40′W). However, no growth was obtained from any of these incubations (Gazert, 1903; Pirie, 1912).

Although bacteria were found in Antarctica in the early 1900s, it took several decades before researchers not only isolated, but also identified Antarctic bacteria. In 1941, Darling and Siple isolated 178 bacteria from Antarctic ice, water, soil, plant debris and snow (Darling and Siple, 1941). Using the techniques available at the time, these bacteria were identified as members of the genera *Bacillus, Achromobacter, Flavobacterium, Micrococcus, Serratia*, and *Proactinomyces* (later reclassified as *Nocardia*).

Contrary to the early discovery of bacteria in Antarctica, archaea were reported from the continent from the late 1980s on. The first Antarctic archaeon was described as a bacterium, since the kingdom of Archaea (originally named Archaebacteria) was only proposed in 1977 (Woese and Fox, 1977). While investigating microorganisms inhabiting Deep lake (68°34′S 78°12′E), Franzmann et al. (1988) isolated the novel species *Halobacterium lacusprofundi*. Although originally described as a bacterium, this organism was later reclassified as an archaeon. More recently, with the use of 16S rRNA gene analysis and later on deep-sequencing techniques, many novel Antarctic prokaryotes have been discovered, although the majority of these remain part of the uncultivated majority.

## Prokaryotic Diversity in Antarctic Ice-Free Soils Based on Cultivation-Independent Studies

To give an update on bacterial diversity found in exposed Antarctic soils, we focus first on studies using cultivation-independent methods and then on results obtained from cultivation. Antarctic edaphic microbial diversity, ecology and biogeochemistry has been previously extensively reviewed (Hogg et al., 2006; Cary et al., 2010; Cowan et al., 2014; Chown et al., 2015; Chong et al., 2015; Makhalanyane et al., 2016, 2017; Pearce, 2017). In this section we therefore highlight a few specific recent findings from cultivation-independent studies which stimulate discussion of fundamental aspects of microbial diversity and ecology in Antarctic soils. We first discuss bacterial diversity and then address data for Archaea at the end of this section.

Cultivation-independent techniques have expanded our understanding of Antarctic bacterial diversity, with one of the most significant revelations being the detection of a much greater microbial biomass and diversity within Antarctic soils than previously predicted (e.g., Cowan et al., 2002; Chong et al., 2012b; Lee et al., 2012). Contrary to other extreme environments, Antarctic and other cold (polar and alpine) soils seem to exhibit a relatively high heterogeneity and appear to be dominated by bacterial lineages (Cowan et al., 2015). While the phylum-level composition does not differ much from those recorded in temperate soils, with Actinobacteria, Acidobacteria, Proteobacteria, and Bacteroidetes being the most dominant phyla, communities seem to be highly specialized at the genus and species level with relatively low numbers of dominant species, and a high diversity of subdominant species (Janssen, 2006; Pointing et al., 2009; Chong et al., 2015; Delgado-Baquerizo et al., 2018). Numerous different genera within 40–70 phyla have been detected in these soils, the exact number being hard to determine due to the general lack of an official classification and nomenclature framework for the many uncultured taxa (Yarza et al., 2014; Konstantinidis et al., 2017). The existence of several alternative classifications and nomenclatures (Supplementary Table S1) complicates comparison between studies and is one of the important challenges in non-cultivation based surveys. Two examples specifically related to Antarctic soil environments are candidate phylum Dormibacteraeota (AD3) and Abditibacteriota (FBP). The former being classified as Chloroflexi in SILVA release 132 (Quast et al., 2013; Yilmaz et al., 2014), and the latter as Unclassified Bacteria in the RDP 11 reference database (Cole et al., 2014). This is important, considering it has been proposed that AD3 bacteria support primary production in Antarctic desert soils, and that Abditibacteriota are abundant in granite soil samples with high total organic carbon content (Tytgat et al., 2016; Ji et al., 2017). To complicate matters even more, the proposed superphylum Patescibacteria (Rinke et al., 2013) includes several candidate phyla (Microgenomates, Parcubacteria, and Saccharibacteria) which themselves have actually been suggested to be superphyla (Rinke et al., 2013; Sekiguchi et al., 2015). However, Patescibacteria is listed in specific sequence databases such as SILVA 132 as a phylum level lineage. Examples like these represent a potential limitation for better understanding of biogeographic patterns, biogeochemistry and systems ecology. These considerations are not trivial for candidate phyla organisms, which arguably represent the majority of all Bacteria and Archaea (Castelle and Banfield, 2018).

Despite the challenges of taxonomic classification, it has become clear that a large fraction of the detected organisms are novel lineages. Studies have shown that up to 80% of bacteria identified from Antarctic soils are yet-to-be cultured, including phylotypes from well-studied phyla such as the Actinobacteria (Smith et al., 2006; Babalola et al., 2009; Lee et al., 2012).

Interesting questions that have risen from this observation are how such diverse communities are able to exist in some of the most extreme oligotrophic environments on Earth, and what the basis of their primary production is. Given that the abundances of classical primary producers such as Cyanobacteria and algae varies greatly across these Antarctic soils, with multiple studies reporting sites at which they were only present in low abundances or nearly absent (Yergeau et al., 2007; Cary et al., 2010; Tytgat et al., 2016), other microorganisms must be responsible for primary production. It should be noted, however, that these discrepancies might also (partially) be the result of methodological artifacts inherent to these methods (Bergmann et al., 2011; Vandeputte et al., 2017; Pollock et al., 2018), and thus care is needed when interpreting limited or non-detection of these groups as absence.

Several recent studies revealed that, in addition to Cyanobacteria, a large diversity of other microorganisms present in these environments may be capable of fixing carbon dioxide (Chan et al., 2013; Niederberger et al., 2015; Tebo et al., 2015; Wei et al., 2015; Tahon et al., 2016a, 2018b; Ji et al., 2017). Analysis of the large subunit of type I ribulose-1,5-biphosphate carboxylase/oxygenase genes (*cbbL*) revealed RuBisCO types of the phyla Proteobacteria, Actinobacteria, WPS-2 and AD3 (Chan et al., 2013; Niederberger et al., 2015; Tebo et al., 2015; Wei et al., 2015; Tahon et al., 2016a, 2018b; Ji et al., 2017), suggesting that diverse bacteria capable of assimilating carbon dioxide through the Calvin-Benson-Bassham cycle. The energy for this primary production may be provided by scavenging atmospheric trace gases H_2_ and CO: combined results from the Windmill islands, Adams Flat, and the McMurdo Dry Valleys provided evidence that bacteria from the phyla Actinobacteria, AD3, and WPS-2 use these processes for chemoautotrophic growth (Ji et al., 2017). Moreover, the ability to persist on atmospheric hydrogen and carbon monoxide has also been detected in Acidobacteria (Greening et al., 2015) and Chloroflexi (Greening et al., 2016; Islam et al., 2018), both of which contain many uncharacterized phylotypes that frequently occur in Antarctic and Arctic soil habitats. Light is an alternative energy source that may be used by a diverse range of non-cyanobacterial taxa in Antarctic soils. Two main mechanisms of light-harvesting have been described in prokaryotes, using either rhodopsins or complex photochemical reaction centers that contain (bacterio)chlorophyll (Bryant and Frigaard, 2006). A screening of several Antarctic soils using clone libraries and Illumina MiSeq sequencing revealed a large sequence diversity of genes involved in bacteriochlorophyll synthesis, including novel sequence homologs (Tahon et al., 2016a,b). Proteo- and actinorhodopsins could, however, not be retrieved from these soils. This does not necessarily imply that rhodopsins are absent in terrestrial Antarctic prokaryotic communities as the available primers used may be unsuitable to capture genes encoding these systems in this environment.

Another enduring question is which members of the community recovered by cultivation-independent techniques, such as 16S rRNA gene amplicon sequencing and metagenomics, are active. In this regard, recent studies have identified Proteobacteria and Deinococcus-Thermus as bacterial phyla that are active, thriving and adapting to environmental conditions in the McMurdo Dry Valleys, while Acidobacteria and Bacteroidetes are believed to be largely inactive. Evidence for this has been provided by using stable isotope probing (Schwartz et al., 2014), metatranscriptomics (Buelow et al., 2016) and 16S rRNA gene amplicon sequencing (Feeser et al., 2018). These studies suggest that Acidobacteria, one of the major phyla recovered from terrestrial ecosystems in Antarctica (Ji et al., 2017; Van Goethem et al., 2018), might persist partially in a variety of dormant forms. In addition, conditions in these dry and cold soils are not very different from those routinely used for the preservation of microbial cultures and microbiomes (Kerckhof et al., 2014; Makhalanyane et al., 2017). A recent study on temperate soils suggests that dead non-intact cells and extracellular genomic DNA may inflate microbial diversity and obscure estimations of taxon relative abundances (Carini et al., 2016). The authors found that the effects of relic DNA removal on estimated relative abundances often approached or even exceeded a 25% increase or decrease, and varied depending on the taxon and soil in question. At some sites, specific taxa such as Actinobacteria and Alphaproteobacteria significantly increased in relative abundance after relic DNA was removed, while Verrucomicrobia decreased. In a small comparison of DNA extraction methods, Tahon et al. (2018b) also noted varying differences in relative abundances of 16S rRNA gene types of different phyla when comparing the use of the PowerLyzer PowerSoil DNA isolation kit with a method that involved removal of external DNA. To date, the extent to which historical community compositions are reflected in current Antarctic soil surveys has not yet been evaluated, but one might hypothesize that the effect is even greater in such cold and dry conditions where dead cells and their released nucleic acids degrade more slowly (Tow and Cowan, 2005).

Recently, the dominance, in terms of their abundances, of taxa from uncultured lineages in nearly all environments on Earth has been estimated by comparing environmental 16S rRNA genes from databases to those of cultured type strains (Lloyd et al., 2018). The authors defined a new concept for bacteria from orders or higher taxa that do not have cultured representatives: phylogenetically divergent non-cultured cells, which unlike viable but non-culturable cells, are microorganisms for which traditional isolation techniques may never succeed. Lloyd et al. (2018) estimate that these phylogenetically divergent non-cultured cells represent the majority of Earth’s microorganisms, and conclude that cultivation-independent methods combined with innovative culturing concepts are needed to gain insights into the physiology and ecology of these abundant and divergent organisms. For soil environments, metagenome-based estimates suggest that 82% of microbial cells are from uncultured genera or higher. Although the specific types of soil sub-environments they analyzed included bogs/fens, permafrost and deserts, it would be interesting to have an estimation of the abundance of uncultured bacteria in Antarctic soil environments specifically. Therefore, we repeated the analysis of Lloyd et al. (2018) specifically using bacterial sequences obtained from Silva 132 Parc ^2^ (Pruesse et al., 2007), which contains primer-amplified, high-quality, >300 bp 16S rRNA gene sequences. The analysis included 68,377 sequences from 37 studies representing both cultivation-dependent and -independent Antarctic soil surveys. The results suggest that, after chimera filtering, 85.6% of bacterial sequences in Antarctic soil environments are from uncultured genera or higher. Considering that primer-amplified databases skew toward cultured organisms (Eloe-Fadrosh et al., 2016; Karst et al., 2018; Lloyd et al., 2018), the abundance of uncultured cells in Antarctic soil environments is indeed probably even much higher than the 82% reported for soil environments in general by Lloyd et al. However, it should be noted that high-throughput sequencing, although often presented as quantitative, is in fact inherently relative (Gloor et al., 2017; Vandeputte et al., 2017). Therefore, care is needed when interpreting these results.

One of these phylogenetically divergent uncultured groups often encountered in Antarctic cold desert mineral soils as one of the most dominant taxonomic units consists of members of the *Ellin6075* family (Acidobacteria). Phylotypes of this uncharacterized lineage have been identified as some of the most dominant (i.e., most abundant and ubiquitous) soil bacterial organisms worldwide (Delgado-Baquerizo et al., 2018). Acidobacteria is just one of the phyla that has resisted substantial cultivation efforts. Indeed, in many studies few of the predominant actinobacterial sequences recovered from Antarctic soils can be matched to cultivated phylotypes at >96.6% homology (Smith et al., 2006; Babalola et al., 2009). The majority represent uncultured taxa most closely related to uncultured Thermoleophilia, *Crossiella*, Nocardioidaceae, Frankiales, Acidimicrobiia and divergent Actinobacteria phylotypes of which the lower taxonomic affiliations remain unclear (Chong et al., 2012a; Aislabie et al., 2013; Ji et al., 2015; Rampelotto et al., 2015; Tytgat et al., 2016; Pudasaini et al., 2017). Other divergent uncultured phylotypes that often constitute a significant fraction of Antarctic soils are Cyanobacteria. These include phylotypes most closely related to different classes such as Leptolyngbyaceae, Nostocaceae, Synechococcaceae, Osciallatoriaceae, Microcoleaceae and Chroococcidiopsidaceae, and other divergent Cyanobacteria of which the lower taxonomic affiliations remain unclear (Jungblut et al., 2010; Chrismas et al., 2015; Makhalanyane et al., 2015; Obbels et al., 2016; Pushkareva et al., 2018; Van Goethem and Cowan, 2019). Abundant uncultured Antarctic Bacteroidetes, Proteobacteria and Chloroflexi include phylotypes most closely related to uncharacterized Cytophagales, Sphingobacteriales, Saprospirales, Acetobacterales, Ktedonobacterales and other divergent sequences with unspecified taxonomic affiliations (Aislabie et al., 2006; Bajerski and Wagner, 2013; Boetius et al., 2015; Tebo et al., 2015; Makhalanyane et al., 2017). Interestingly, uncultivated Ktedonobacterales and other uncharacterized Chloroflexi representatives are among the most dominant groups recovered from high altitude sites in the Atacama desert (Lynch et al., 2012) and permafrost soils worldwide (Jansson and Taş 2014; Tuorto et al., 2014), suggesting these might represent uncharacterized lineages adapted to extreme oligotrophic environments. Moreover, it has been suggested that the class Ktedonobacteria should be regarded as a lineage related to Chloroflexi *sensu stricto*, and that at present Chloroflexi constitutes a superphylum (Gupta et al., 2013).

In addition to uncultured representatives of the most intensively studied phyla, members of a number of uncultivated candidate phyla, and phyla that until recently only consisted of uncultured representatives are often present in Antarctic soil data sets. The most abundant include Dormibacteraeota (AD3), Eremobacteraeota (WPS-2), Patescibacteria (most notably Saccharibacteria, Kazan, WS5, Microgenomates and Berkelbacteria) and Abditibacteriota (Quast et al., 2013; Winsley et al., 2014; Ji et al., 2015; Tytgat et al., 2016). For many of these uncharacterized taxa, the question remains what their role and significance is in biogeochemical processes that impact Antarctic soils and atmospheric chemistry. Considering some of these soils and Antarctic regions are shown to be among the most rapidly warming areas of the planet (Steig et al., 2009; Bromwich et al., 2013; Mohajerani et al., 2018), and that climate change will lead to pronounced shifts in the diversity of soil bacteria (Ladau et al., 2018), with stronger warming effects on microbial abundances in colder regions (Chen et al., 2015), additional and more sequence surveys, as well as physiological studies providing more comprehensive data, are needed (see below).

Presently, Archaea are the least studied members of Antarctic soil microbial communities, with very few surveys focusing specifically on this domain of life. Studies have consistently reported a low abundance and diversity of Archaea in Antarctic soils, with the majority of sequences (80–99%) affiliated with the ammonia-oxidizing Thaumarchaeota (formerly known as Crenarchaeota Marine Group 1.1b) (Ayton et al., 2010; Richter et al., 2014). A notable exception being the study of Pessi et al. (2015), where Euryarchaeota has been recorded as the dominant archaeal phylum and the third most abundant group in recently exposed soil from the Wanda Glacier forefield on King George island (Pessi et al., 2015). As most of these studies relied on amplicon sequencing of 16S rRNA genes, it is possible that the primers used failed to capture the full range of archaeal diversity. Indeed, recent results from various environments suggest that archaeal diversity has been largely overlooked due to the limitations of available primers, and that improved primer pairs enable a more comprehensive estimation of archaeal diversity and the discovery of novel archaeal taxa at various phylogenetic levels (Bahram et al., 2018). Very few metagenome studies, where total environmental DNA is sequenced without prior amplification, have been reported from Antarctic soils. Pearce et al. (2012) reported Euryarchaeota, Crenarchaeota and Korarchaeota, however no Thaumarchaeota in a soil community at Mars Oasis on Alexander Island. More recently Le et al. (2016) reported 0.43% archaeal phylotyes in hypolithic communities from Miers Valley. Halobacteria and Methanomicrobia (phylum Euryarchaeota) were the predominant groups, while Crenarchaeota, Thaumarchaeota and Korachaeota were also recovered. For all these groups a portion of the sequences remained unclassified at higher levels (Le et al., 2016). In addition to the aforementioned archaeal phyla, metagenome datasets from Antarctic Dry Valley soil that are available at JGI-IMG/M (Markowitz et al., 2014) comprise phyla including several Candidatus phyla that belong to the DPANN superphylum (Hug et al., 2016). Therefore, it is reasonable to presume that the exact diversity and role of Archaea in Antarctic edaphic habitats has yet to be resolved. Recent studies have highlighted the importance of ammonia-oxidizing archaea (Thaumarchaeota) in the nitrogen cycle of soils in the Antarctic Peninsula (Jung et al., 2011) and in the McMurdo Dry Valleys (Magalhaes et al., 2014). Also, the first genome of a novel uncultured methane-producing *Methanosarcina* species has been reported from 15,000-year-old permafrost from Miers Valley (Vishnivetskaya et al., 2018). These Antarctic methanogens might provide positive feedbacks to climate change when near-surface permafrost thaws and the active soil layer deepens.

## Cultured Prokaryotic Diversity in Terrestrial Antarctic Ecosystems

From the first half of the 20th century onward, several researchers have attempted to isolate prokaryotes and in particular bacteria from Antarctic soils. To our knowledge, no archaea have been isolated from Antarctic ice-free areas to date. Therefore, the focus of this part will be on cultivated bacterial diversity.

**FIGURE 1:**
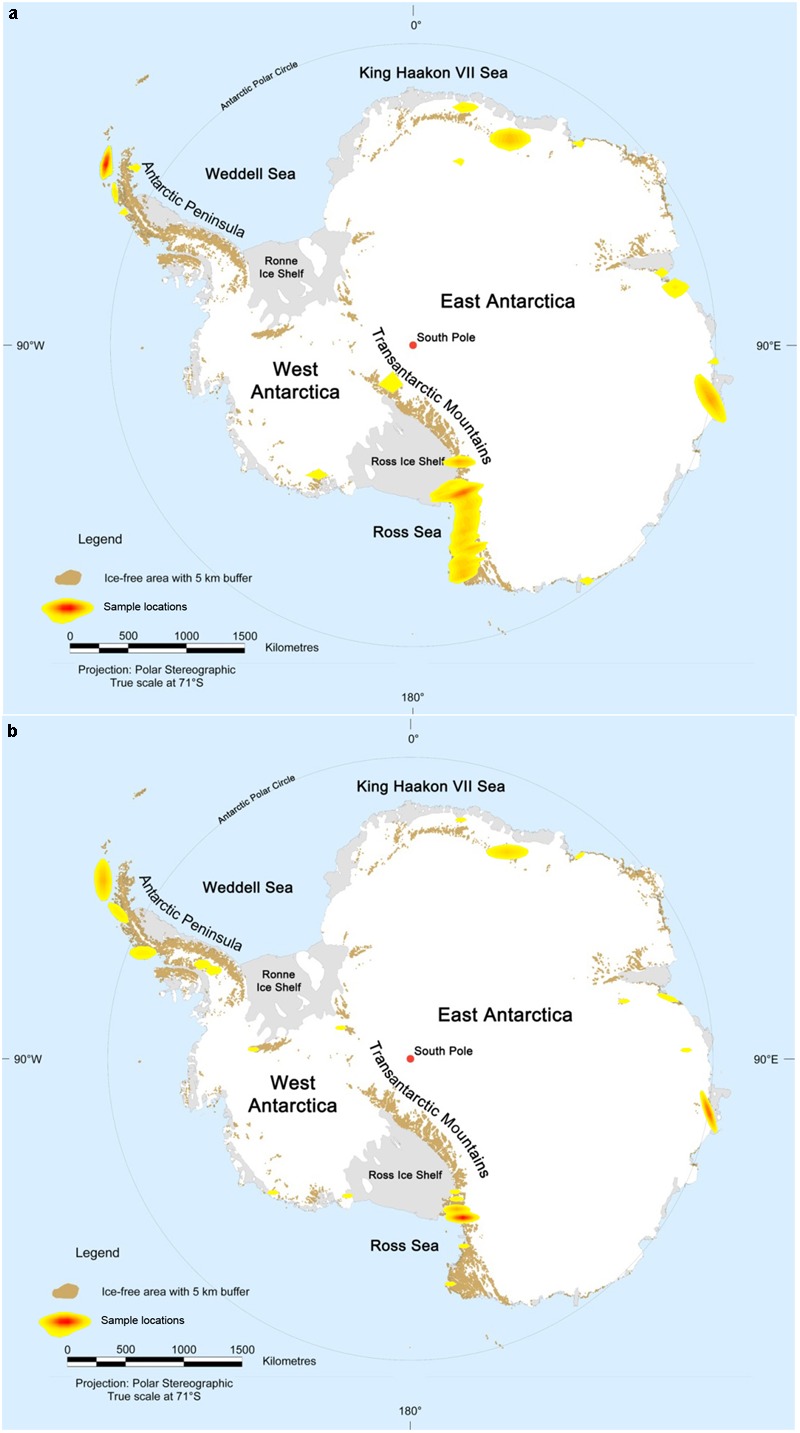
Overview of the Antarctic continent showing ice-free regions sampled for bacterial diversity studies using **(a)** cultivation (84 studies, 156 samples) and **(b)** cultivation-independent (105 studies, 1961 samples) surveys. Ice-free areas are highlighted in brown. Sampling sites are shown in a color gradient from yellow to red with increasing number of sample locations. Map modified from map no. 13766 of the Australian Antarctic Data Centre (2010).

When mapping the sampling locations of such studies, it becomes clear that only a very limited number of sites, comprising a small percentage of the ∼50,000 km^2^ of exposed Antarctic soils, have been investigated (Figure 1a). Similar observations can be made for cultivation-independent surveys, although differences exist between the investigated areas (Figure 1a,b). The regions which have received most attention over the years are King George Island and Victoria Land. Because of its northern position, King George Island is relatively easily accessible compared to the rest of the continent. Over the years, this small island has hosted more than ten research stations, explaining the large number of studies carried out here. Victoria Land, on the other hand, is located in Eastern Antarctica, extending southward from ∼70°30′S to 78°00′S, and westward from ∼160°E to 170°E (Wall et al., 2006). Although the culturable microbial diversity has been investigated in terrestrial sites scattered throughout this region, the majority of studies focused on soils of the McMurdo Dry Valleys, located in the northern part of Victoria Land and considered as one of the most extreme deserts on Earth (Barrett et al., 2006). In addition, several research groups have also assessed soils from other coastal regions, while a few studies have focused on more inland ice-free regions, such as the Sør Rondane Mountains or the Vestfold Hills (Smith et al., 2006; Peeters et al., 2011; Lepane et al., 2018).

**FIGURE 2:**
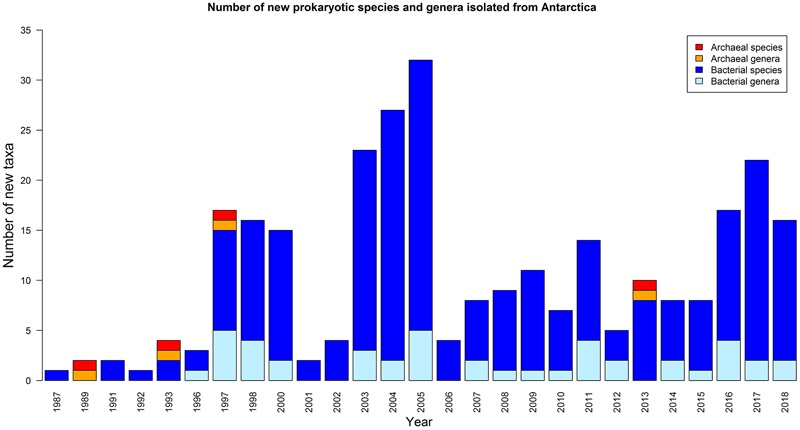
Overview of the number of newly described prokaryotic species and genera isolated from Antarctica based on articles included in Web of Science (up to 16 January 2019).

Depending on the research hypothesis investigated, many different cultivation approaches have been used, to target either a broad variety or a particular subset of the bacteria inhabiting the Antarctic soils. While over the past 30 years, a variety of novel taxa have been described from various Antarctic environments (Figure 2, Table 1, and Supplementary Table S2), isolation campaigns have recovered members of only six bacterial phyla, i.e., Proteobacteria, Cyanobacteria, Actinobacteria, Deinococcus-Thermus, Bacteroidetes and Firmicutes, while there are 33 phyla with cultivated representatives as of January 2019^3^. Together, these six phyla encompass a large portion of currently cultured and sequenced diversity. However, only a small number of genera from these phyla have been cultured from Antarctic soils (i.e., ∼110). Several of the groups frequently recovered during isolation campaigns from Antarctic soils have been validly described as novel Antarctic species or genera (Table 1). Apart from these, many other genera have been cultivated from Antarctic soils, although often in lower abundances (Table 2). Additionally, bacterial isolates that very likely represent the first cultured representative of novel as yet unnamed genera have been picked up during several isolation campaigns involving Antarctic soils (Peeters et al., 2011; Pulschen et al., 2017; Tahon and Willems, 2017).

**Table 1:** List of prokaryotic genera that contain validly described species originating from Antarctica.

Genus			No. of species	Genus			No. of species
Archaea				Cyanobacteria			
Euryarchaeota				^∗^	*Aliterella*		2
^∗^	*Halohasta*		1	Deinococcus-Thermus			
^∗^	*Halorubrum*		1	†	*Deinococcus*		6
^∗^	*Methanococcoides*		1	Firmicutes			
^∗^	*Methanogenium*		1	†	*Alicyclobacillus*		1
				†	*Aneurinibacillus*		1
Bacteria				†	*Anoxybacillus*		1
Abditibacteriota				†	*Bacillus*		3
^∗^†	*Abditibacterium*		1	†	*Brevibacillus*		1
Actinobacteria				†	*Carnobacterium*		4
	*Amycolatopsis*		1		*Clostridium*		6
†	*Arthrobacter*		9	†	*Exiguobacterium*		2
^∗^†	*Friedmanniella*		2	†	*Paenibacillus*		4
†	*Kocuria*		1	†	*Planococcus*		3
†	*Leifsonia*		5	^∗^	*Psychrosinus*		1
^∗^	*Marisediminicola*		1	†	*Sporosarcina*		2
†	*Micrococcus*		1	†	*Staphylococcus*		1
†	*Micromonospora*		1	Proteobacteria			
^∗^†	*Modestobacter*		1	^∗^	*Actimicrobium*		1
†	*Nesterenkonia*		2	^∗^	*Allohahella*		1
†	*Nocardioides*		1		*Alteromonas*		1
†	*Pseudonocardia*		1		*Campylobacter*		1
^∗^†	*Raineyella*		1		*Colwellia*		4
†	*Rhodococcus*		1	^∗^†	*Constrictibacter*		1
^∗^	*Rhodoglobus*		1		*Desulfovibrio*		1
	*Sanguibacter*		2	^∗^	*Glaciecola*		2
†	*Streptomyces*		2	^∗^	*Granulosicoccus*		1
Bacteroidetes					*Hahella*		1
	*Aequorivita*		1		*Halomonas*		2
	*Algoriphagus*		1		*Kiloniella*		1
^∗^	*Antarcticibacterium*		1	^∗^	*Loktanella*		3
^∗^	*Antarcticimonas*		1	†	*Lysobacter*		1
^∗^	*Aquaticitalea*		1		*Marinobacter*		3
	*Arenibacter*		1		*Marinomonas*		1
	*Bizionia*		5	^∗^	*Methylosphaera*		1
	*Cellulophaga*		1		*Neptunomonas*		1
^∗^	*Changchengzhania*		1		*Oceanicola*		1
†	*Flavitalea*		1	^∗^	*Octadecabacter*		1
†	*Flavobacterium*		25	^∗^	*Oleispira*		1
	*Flectobacillus*		1		*Pararhizobium*		1
^∗^	*Gelidibacter*		3	^∗^†	*Polaromonas*		1
^∗^	*Gillisia*		4	^∗^†	*Polymorphobacter*		1
	*Gramella*		1		*Pseudoalteromonas*		3
^∗^†	*Hymenobacter*		6	†	*Pseudomonas*		10
	*Kordia*		1	^∗^	*Pseudooceanicola*		1
^∗^	*Lacinutrix*		2		*Pseudorhodobacter*		1
	*Lewinella*		1		*Psychrobacter*		9
	*Maribacter*		1	^∗^†	*Psychromonas*		2
†	*Mucilaginibacter*		2	^∗^	*Puniceibacterium*		1
	*Muricauda*		1	†	*Rhodoferax*		1
	*Nonlabens*		1	^∗^	*Rhodoligotrophos*		1
†	*Pedobacter*		5	^∗^	*Robiginftomaculum*		1
	*Polaribacter*		1	^∗^	*Roseicitreum*		1
^∗^	*Pricia*		1	^∗^	*Roseisalinus*		1
^∗^	*Psychroflexus*		2		*Roseovarius*		1
^∗^	*Psychroserpens*		2	^∗^	*Saccharospirillum*		1
^∗^†	*Sejongia*		3		*Shewanella*		4
†	*Sphingobacterium*		1	†	*Sphingomonas*		4
^∗^	*Subsaxibacter*		1	^∗^	*Staleya*		1
^∗^	*Subsaximicrobium*		2		*Sulfitobacter*		1
	*Ulvibacter*		1		*Thalassospira*		1
^∗^	*Williamwhitmania*		1	^∗^	*Zhongshania*		2

*Genera first described from Antarctica are marked with a ^∗^. Genera recovered during isolation campaigns involving Antarctic soil are marked with a †. A detailed list of the species involved is provided in Supplementary Table S2.*

**Table 2:** Bacterial genera that were recovered during isolation campaigns from Antarctic soil, for which, however, no validly named Antarctic species have been described.

Genus	Genus	Genus	Genus
*Acidothermus*	*Burkholderia*	*Massilia*	*Rothia*
*Acinetobacter*	*Caulobacter*	*Methylobacterium*	*Ryzobacter*
*Adhaeribacter*	*Conexibacter*	*Microbacterium*	*Saxeibacter*
*Aeromicrobium*	*Cryobacterium*	*Motilibacter*	*Schumannella*
*Aminobacter*	*Dermacoccus*	*Mycobacterium*	*Sphingobium*
*Angustibacter*	*Devosia*	*Nakamurella*	*Sphingopyxis*
*Aquaspirillum*	*Dyella*	*Nostoc*	*Spirosoma*
*Aquipuribacter*	*Frankia*	*Noviherbaspirillum*	*Stenotrophomonas*
*Aurantimonas*	*Frondihabitans*	*Paenisporosarcina*	*Subtercola*
*Auraticoccus*	*Geodermatophilus*	*Paracraurococcus*	*Terrabacter*
*Aureimonas*	*Janthinobacterium*	*Patulibacter*	*Tessaracoccus*
*Azospirillum*	*Kineosporia*	*Phycicoccus*	*Tetrasphaera*
*Beijerinckia*	*Knoellia*	*Quadrisphaera*	*Thalassiella*
*Brachybacterium*	*Kribbella*	*Rhizobium*	*Variovorax*
*Bradyrhizobium*	*Lapillicoccus*	*Rhodophila*	*Xylophilus*
*Brevibacterium*	*Lysinibacillus*	*Rhodopseudomonas*	
*Brevundimonas*	*Marmoricola*	*Roseomonas*	

More recently, one isolate, representing the first cultivated member of the Abditibacteriota (formerly known as candidate phylum FBP), was recovered from soils of the Sør Rondane Mountains, East Antarctica (Tahon et al., 2018a). On the other hand, cultivation-independent approaches have shown that, apart from the six aforementioned phyla, members of the other phyla with non-Antarctic cultivated representatives are also present in Antarctic terrestrial ecosystems, according to publicly available metagenomes in MG-RAST (Wilke et al., 2016) and IMG-M (Markowitz et al., 2014).

Comparing results from culture-dependent and culture-independent studies on Antarctic soils has confirmed that these environments harbor an enormous amount of microbial dark matter. Although from the late 1980s onward several research groups have isolated and described new bacterial species from Antarctica, a clear rise in the number of newly described species is not apparent (Figure 2). This is in contrast to increasing bacterial species descriptions in general (Sutcliffe et al., 2012) and is probably largely due to the cost of obtaining samples from Antarctica. Furthermore, it should be noted that various studies conducted in the Antarctic did not focus on bacterial diversity but rather on bacterial functions (O’Brien et al., 2004; Lo Giudice et al., 2007; Gesheva and Vasileva-Tonkova, 2012; Arenas et al., 2014; Gran-Scheuch et al., 2017). During these studies, large numbers of isolates from different experiments were subjected to various screenings (e.g., phenanthrene degradation, antimicrobial activity) after which only those giving positive results were used for further analyses and taxonomic identification.

Most of the described Antarctic species group within just four phyla, which are also recovered in cultivation-based surveys worldwide. The majority of recent Antarctic cultivation-based studies use classical approaches (i.e., general growth media, aerobic headspace, ambient temperature, short incubation periods) (O’Brien et al., 2004; García-Echauri et al., 2011; Tomova et al., 2014; Vasileva-Tonkova et al., 2014; Antony et al., 2016; González-Rocha et al., 2017; Gran-Scheuch et al., 2017; Pudasaini et al., 2017). Although as a result multiple novel species have been described from Antarctica, these mostly belonged to the same limited number of phyla (Table 1 and Supplementary Table S2). Nevertheless, by using a wider diversity of cultivation techniques and conditions, it is possible to cultivate a larger diversity of bacteria and reduce the amount of microbial dark matter. This was shown in recent studies that introduced relatively simple changes and succeeded in cultivating novel isolates from previously uncultivated groups. The first member of the Abditibacteriota, isolated from a soil sample of the Sør Rondane Mountains, was observed as a microcolony after 10 weeks of incubation on a low-nutrient medium (Tahon et al., 2018a). During this isolation campaign, with incubation at 4 and 15°C for up to 18 months, several other strains with low 16S rRNA gene sequence similarity with any other known cultured species, and thus very likely to be novel taxa, were also isolated (Tahon and Willems, 2017). Many of these were also isolated as very slow-growing microcolonies, and are nearly invisible to the naked eye. Using a similar approach of low nutrient media and incubation periods up to 15 weeks, Pulschen et al. (2017) isolated many potentially novel taxa from soil sampled on King George Island. Many failed to grow on R2A, similar to many of the isolates reported by Tahon and Willems (2017). While the low-nutrient R2A medium is commonly used for bacterial isolation from cold environmental samples (Pulschen et al., 2017), these recent studies suggest that many novel isolates may require even more oligotrophic conditions for growth.

Cultivation certainly has many major benefits compared to cultivation-independent approaches. A living culture permits experimental verification of a bacterium’s behavior under certain conditions or stresses (e.g., temperature, antibiotic resistance). It also allows *ex situ* preservation for future studies and applications. Bacteria generally play a major role in today’s society and have done so for a long time in pharmaceutical, biotechnological, bioremediation and food industry applications. In this respect, Antarctic bacteria are very interesting as they have evolved to survive and even thrive in extreme environmental conditions, making them suitable candidates for the screening of potentially interesting novel compounds and enzymes. A number of studies have been carried out to isolate Antarctic soil bacteria and characterize their functional potential. Several studies looked for bacteria with remediation capacities for phenol (Lee et al., 2018) and polycyclic aromatic hydrocarbons (Gran-Scheuch et al., 2017). Lee et al. (2018) observed that among isolates obtained from King George island, two *Arthrobacter* spp. and one *Rhodococcus* sp. were capable of quickly and completely degrading phenol at temperatures between 10 and 15°C. Gran-Scheuch et al. (2017) isolated 47 Antarctic strains exhibiting varying phenanthrene degradation capacities. Three of these, all isolated from diesel contaminated soils and identified as *Rhodococcus erythropolis* D43AFA, *Pseudomonas guineae* E43FB and *Sphingobium xenophagum* D43FB degraded 69, 86 and 95% of the phenanthrene, respectively. While looking for strains with high potential for biodegradation of hydrocarbons, Gesheva and Vasileva-Tonkova (2012) isolated a *Nocardioides* sp. that also produced compounds with antibacterial activity against Gram-positive and Gram-negative bacteria, especially *Staphylococcus aureus* and *Xanthomonas oryzae*. Antimicrobial activity against the latter species may be of great importance given that *X. oryzae* is a plant pathogen causing one of the most harmful diseases of rice (Banerjee et al., 2018). The results of Gesheva and Vasileva-Tonkova (2012) revealed a great potential of the *Nocardioides* strain as a source of industrially important enzymes and antimicrobial compounds for biocontrol, biotechnological and biopharmaceutical applications. Muñoz et al. (2017) found that antifreeze proteins produced as a survival strategy by the Antarctic psychrophiles *Pseudomonas* sp. AFP5.1, *Sphingomonas* sp. GU1.7.1 and *Plantibacter* sp. GU3.1.1 protect cellular structures of frozen food and therefore could be very useful for the food industry. O’Brien et al. (2004) showed that several Antarctic bacteria, including *Arthrobacter* spp., *Pseudomonas* spp. and *Planococcus* spp. could be used as growth-inhibitors of different food spoilage or pathogenic bacteria because of their ability to produce cold-active antimicrobial compounds which could be useful for chilled-food preservation. Pigments from UV-resistant Antarctic bacteria such as *Hymenobacter* sp. A9A5(R) and *Chryseobacterium* sp. A9A5(A) may be of interest as photosensitizers for use in Dye Sensitized Solar Cells (Órdenes-Aenishanslins et al., 2016). Many of the aforementioned strains group in genera or species which are commonly found in Antarctic soils (Table 1 and Supplementary Table S2). Overall, these studies show that Antarctic soils represent a largely untapped reservoir of novel, cold-active microorganisms with great potential for industrial, bioremediation and medical applications.

As described above, the diversity of cultivated bacteria in general is very limited, even more so for Antarctic bacteria. Cultivation-based studies do not give an accurate overview of which bacteria or archaea are present in a certain environment, a discrepancy often referred to as the Great Plate Count anomaly (Staley and Konopka, 1985). Given that cultivation involves a choice of medium and growth conditions which are usually limited by practical and budgetary considerations, this is understandable. However, now that uncultivated surveys have highlighted the dominance of a few phyla and the stark lack of cultures from the majority of phyla, the use of more diverse cultivation strategies should be a priority. While so far, a limited number of alternative cultivation conditions have been used, such studies have succeeded to cultivate novel diversity. To increase even more the number of (novel) prokaryotes from Antarctic soils, the use of a range of other, more diverse and unusual cultivation approaches therefore holds great promise (Alain and Querellou, 2009). Given the importance of bacteria in so many processes and aspects of our life, cultivation should be revisited to enlarge the number of bacteria in to culture, making them accessible for scientific study and application.

## Toward a Synergistic Approach

For a very long time, the limitations associated with cultivation-based approaches were unknown. This rapidly changed with the development of different molecular methods that could be used to study prokaryotic diversity without cultivation. Initially the use of clone libraries of 16S rRNA genes clearly showed that only some prokaryotes could be grown and that these were often not the dominant members of the community (Rappe et al., 2002; Janssen, 2006). As high-throughput sequencing techniques and, more recently, long read single molecule sequencing were introduced, 16S rRNA gene amplicon and metagenome sequencing of microbial communities significantly changed the tree of life and revealed large amounts of prokaryotic dark matter (i.e., uncultured prokaryotes). Based on existing sequence data, the prokaryotes are currently estimated to comprise approximately 100,000 species distributed over at least 310 phyla (Brown et al., 2015; Hug et al., 2016; Locey and Lennon, 2016; Parks et al., 2017; Zaremba-Niedzwiedzka et al., 2017; Castelle et al., 2018) and these numbers continue to grow as more data accumulates. Using sequence data for 508 Antarctic soil samples kindly provided by Jeff Bowman (Bowman, 2018), we estimate that Antarctic soil environments contain approximately 8286 and 974 species (97% cutoff) distributed across at least 63 and 11 phyla (based on classification using the silva.nr_v132 database) for Bacteria and Archaea, respectively.

Although most of this newly discovered diversity has never been brought into culture, much can be learned from these large metagenomics datasets. Contrary to cultivation, they give a more comprehensive overview of the community composition in a certain environment. Furthermore, they can provide insight in the functional potential of a community. Recently, advances in sequencing throughput and computational techniques have allowed the recreation of genomes from metagenome data. Although they can be biased by genomic micro-heterogeneity (Nichols, 2007; Prosser, 2015), these analyses have several advantages as they document the genomic potential of prokaryotic phyla previously known only by their 16S rRNA gene sequences, and thus provide insight in microbial dark matter (Parks et al., 2017). Nevertheless, such data alone does not indicate when and how an organism or a community will perform a certain function. To address these questions, techniques such as metatranscriptomics or metaproteomics can be used. While metagenomics tells us which prokaryotes are present and what genomic potential these organisms have, metatranscriptomics and metaproteomics can allow more insight in their activity by elucidating which genes are being differentially transcribed or expressed (Franzosa et al., 2014; Bashiardes et al., 2016; Petriz and Franco, 2017). This way, active metabolic pathways can potentially be identified and associated to certain environmental conditions (Bikel et al., 2015). Assigning functions detected in a community to particular prokaryotes depends on correct binning of sequence data which can be a challenge even in the relatively low complexity soils (Gutleben et al., 2018) of Antarctica.

On the other hand, while cultivation and non-cultivation based surveys each reveal a distinct portion of the prokaryotic diversity present in the investigated sample, the overlap between the diversity retrieved is often small. This was shown for soils from an apple orchard where cultivation revealed 453 OTUs in common with an uncultivated survey and 601 OTUs were only recovered by cultivation and thus belonged to the rare community members (Shade et al., 2012). For Antarctic soils, a cultivation study for aerobic anoxygenic phototrophic bacteria yielded 77 unique isolates of which 47 were not recovered by 16S rRNA gene amplicon sequencing of community DNA (Tahon and Willems, 2017). Furthermore, a genus-level comparison of cultivated heterotrophic bacteria from exposed soil samples with pyrosequencing 16S rRNA gene data revealed that 25.6% of the genera identified by cultivation were not detected by pyrosequencing (Tytgat et al., 2014).

Meta-omics approaches are considered state of the art for analyzing prokaryotic communities in environmental samples. These techniques provide millions of sequences that, after thorough quality control and assembly, lead to a more comprehensive picture of the microbial diversity in the investigated sample. Although meta-omics are revolutionizing our knowledge of microbial life, results obtained using these approaches would greatly benefit from increased cultivation. One of the main limitations of meta-omics analyses is that a large portion of genes can only be assigned a hypothetical function or no function at all (Huynen et al., 2000; Ellrott et al., 2010). Although several hypothetical genes are highly conserved, they still remain without an assigned function because they have either not been studied experimentally or because experimental studies resulted in contradictory results (Galperin and Koonin, 2010). Cultivation followed by genome sequence analysis and experimental testing of the organism’s capabilities in pure or mixed cultures could help to reduce the number of unassigned or hypothetical genes. The data can be used to expand and improve sequence databases that will facilitate improved annotation of future omics studies (Gutleben et al., 2018).

As discussed above, traditional cultivation approaches alone will not lead to a tremendous increase in cultivated prokaryotic diversity. In the environment, large amounts of microbial dark matter are present and contribute to the functioning of the community (Lloyd et al., 2018). Each of these organisms has their own specific needs for growth and functioning. However, without more knowledge on the requirements for growth of this microbial dark matter, cultivating them is a virtually impossible task. Here, data from meta-omics can provide useful insights. In recent years, advances in sample treatment, sequencing platforms, sequencing quality, read length and computational analysis tools have led to the recreation of 1000s of near-complete high-quality MAGs from a variety of environments all over our planet (Wrighton et al., 2012; Sharon et al., 2013; Bowers et al., 2017; Parks et al., 2017; Thrash et al., 2018; Tully et al., 2018; Wilkins et al., 2018). Subsequent annotation and analysis of these MAGs together with analysis of the metatranscriptome provides key information on the (partial) presence and expression of metabolic pathways and can thus provide insights into their potential needs for growth (Nichols et al., 2008; Anantharaman et al., 2013, 2018; Ward et al., 2018). Several software tools have been developed to facilitate such studies [e.g., Traitar, to derive phenotypes from a genome sequence (Weimann et al., 2016); TreeWAS for genome-wide association studies (Collins and Didelot, 2018)]. While no examples are yet available of metagenome-assisted cultivation of novel uncultured groups from Antarctic samples, some of the Antarctic MAGs do provide inspiration for targeted cultivation. For example, MAGs derived from the microbial guilds in soils from Robinson Ridge, Wilkes Land, suggest that selective cultivation for hydrogen oxidation may favor isolation of representatives of AD3, WPS-2, Verrucomicrobia, Chloroflexi and Actinobacteria, while selecting for CO oxidation may allow cultivation of AD3 and Actinobacteria (Ji et al., 2017).

Beyond the advantages of combining the two major approaches for analyzing prokaryotic communities, to cultivate more and especially uncharacterized prokaryotes, new cultivation concepts should be used, especially in Antarctic exposed soils where extreme environmental conditions pose additional challenges. One of these new cultivation concepts that has been recently applied to extreme polar soils is the cryo-iPlate which was tested for cultivation of microorganisms in the Canadian high arctic, an analog to the permafrost terrain observed on Mars (Goordial et al., 2017). The cryo-iPlate was modeled off of the ichip method (Nichols et al., 2010), utilizing diffusion of *in situ* nutrients across a 0.03 μm pore size membrane into solid media (i.e., gellan gum) to form a medium that more closely mimics *in situ* conditions. Microbial recovery with the original ichip has been shown to exceed multifold that achieved by standard cultivation, and many of the species cultivated were found to be of significant phylogenetic novelty. A similar new micro-culturing technique that has been recently applied to subantarctic soil is the soil substrate membrane system (Ferrari et al., 2008). The technique mimics *in situ* growth conditions of the (micro)environment by using environmental soil as the growth substrate, thereby avoiding the excessive nutrients and artificial nature of classical media. The soil substrate membrane system has been shown to significantly increase the culturability of soil bacteria, with reports of up to 76% of culture-independent diversity recovered (Svenning et al., 2003; Ferrari et al., 2005; Rasmussen et al., 2008). The method was used successfully in Antarctic soils by van Dorst et al. (2016). However, these authors suggest that combining the soil substrate membrane system with cell sorting applications such as flow cytometry and micro-manipulation (Miteva and Brenchley, 2005; Ferrari and Gillings, 2009) might be more suitable for cultivating rare members of extreme environments (van Dorst et al., 2016). Although not yet applied for isolation of slow growing psychrophiles, another interesting approach to potentially overcome the limitations and technical difficulties of obtaining pure cultures might be the reconstruction of MAGs from co-cultures or enrichment cultures (Driscoll et al., 2017; Ren et al., 2017) and subsequent mining of the genome for phenotypic features that might help to facilitate isolation (Weimann et al., 2016).

In conclusion, while cultivation of prokaryotes from Antarctic samples has led to the regular description of novel species and genera, these belong to a very limited number of phyla as is also observed in classical cultivation studies from non-Antarctic habitats. This contrasts strongly with the broad diversity of phyla revealed by non-cultivation based community surveys. More diverse isolation conditions, inspired by information gleaned from meta-omics data, using the environment itself to provide unknown but essential components for growth, or co-culture with other microorganisms from the same environment can allow more diverse prokaryotes to be brought into culture, making them accessible for detailed studies. Information from such newly cultured prokaryotes can then enrich public sequence databases and contribute to improving the annotation of future datasets, providing new resources that may lead to novel applications.

## Author Contributions

GT, AW, and SL wrote the paper. All authors approved the final manuscript.

## Conflict of Interest Statement

The authors declare that the research was conducted in the absence of any commercial or financial relationships that could be construed as a potential conflict of interest.

## Footnotes

1 https://bacdive.dsmz.de/

2 www.arb-silva.de

3 http://www.bacterio.net/-classifphyla.html

## Supplementary Material

Figure S1 Overview of the Antarctic continent showing the major geographic regions. Ice-free areas are highlighted in brown (modified from map no. 13.766 of the Australian Antarctic Data Centre, 2010).

Table S1 Names of phylum/superphylum level groups^a^ in different sequence databases^b^.

Table S2 List of valid prokaryotic species that have been described with a type strain originating from Antarctica, listed alphabetically per phylum. Genera first described from Antarctica are marked with a ^∗^. Genera recovered during isolation campaigns involving Antarctic soil are marked with a †.

## Abbreviations

MAG: metagenome-assembled genome
