# Supplementary figures and images for: Uncovering the Uncultivated Majority in Antarctic Soils: Toward a Synergistic Approach
Source: Front Microbiol. 2019 Feb 15;10:242. doi: 10.3389/fmicb.2019.00242 (PMC6385771; doi:10.3389/fmicb.2019.00242)

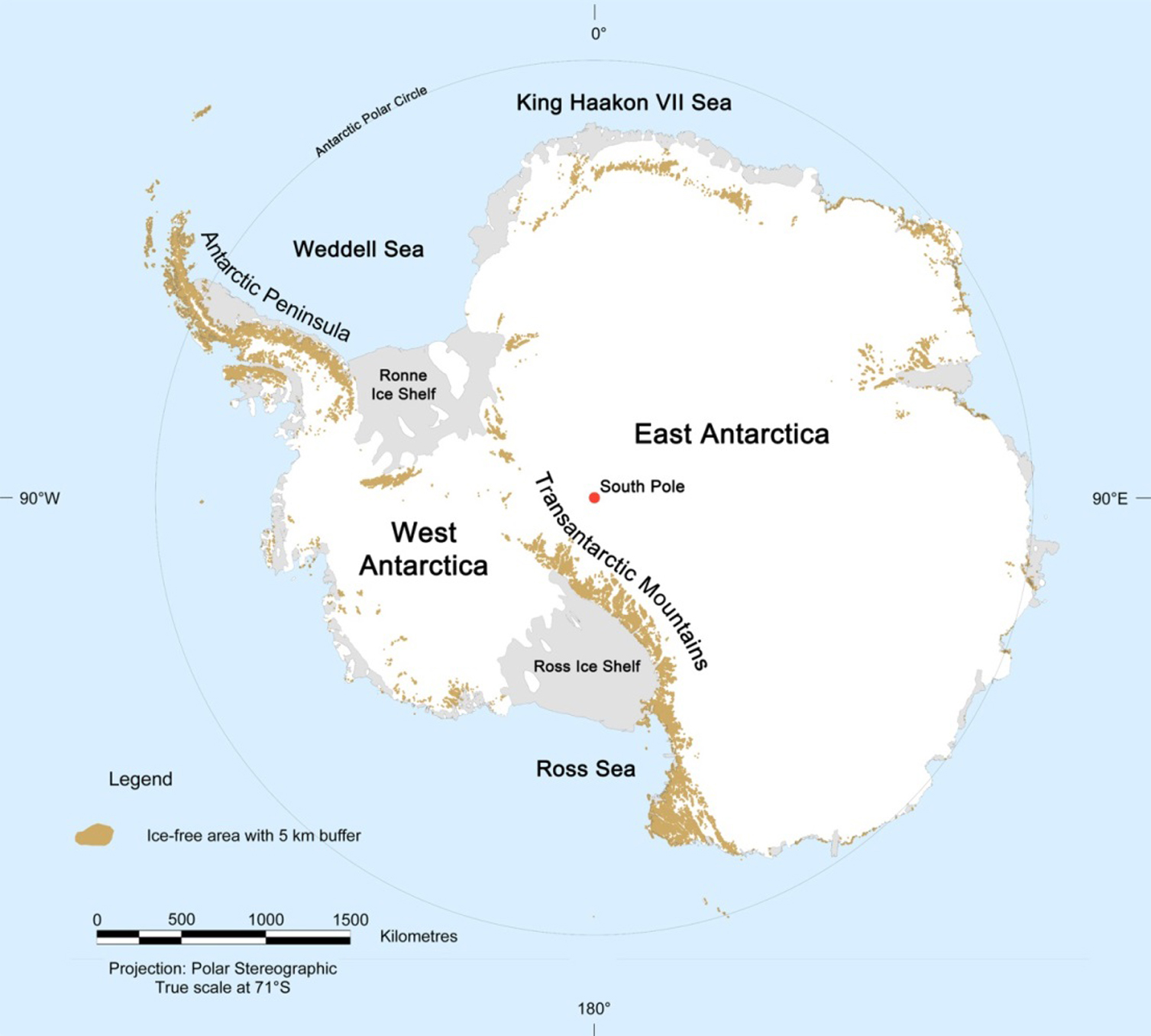

Supplement: Figure S1 — Overview of the Antarctic continent showing the major geographic regions. Ice-free areas are highlighted in brown (modified from map no. 13.766 of the Australian Antarctic Data Centre, 2010). [file Image_1.JPEG]
