# Supplementary material for: Uncovering the Uncultivated Majority in Antarctic Soils: Toward a Synergistic Approach
Source: Front Microbiol. 2019 Feb 15;10:242. doi: 10.3389/fmicb.2019.00242 (PMC6385771; doi:10.3389/fmicb.2019.00242)
Supplement: Table S1 — Names of phylum/superphylum level groupsa in different sequence databasesb. [file Table_1.docx]

Table S1. Names of phylum/superphylum level groups^a^ in different sequence databases^b^

| **SILVA 132** | **Greengenes (Aug 2013)** | **RDP 11** | **NCBI (Taxonomy browser)** | **Alternative names** |
| --- | --- | --- | --- | --- |
| Acetothermia | OP1 | Acetothermia | Candidatus Bipolaricaulota | KB1, OP1, MSBL6, Fraserbacteria |
| Acidobacteria | Acidobacteria | Acidobacteria | Acidobacteria |  |
| Acidobacteria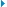Aminicenantia | OP8 | Aminicenantes | Candidatus Aminicenantes | OP8 |
| Acidobacteria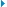Fischerbacteria |  |  | Candidatus Fischerbacteria |  |
| Acidobacteria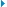Thermoanaerobaculales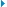Thermoanaerobaculaceae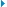TPD-58 | TPD-58 |  | Acidobacteria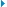Acidobacteria subdivision 23 |  |
| Actinobacteria | Actinobacteria | Actinobacteria | Actinobacteria |  |
| Aegiribacteria |  |  | Candidatus Aegiribacteria | Hyd24-12 |
| Aerophobetes | CD12 |  | Candidatus Aerophobetes | CD12 |
| AncK6 | AncK6 |  |  |  |
| Aquificae | Aquificae | Aquificae | Aquificae |  |
| Armatimonadetes | Armatimonadetes |  | Armatimonadetes | OP10 |
| Atribacteria | OP9 | Atribacteria | Candidatus Atribacteria | JS1, OP9 |
| Bacteriodetes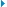Ignavibacteria |  | Ignavibacteriae |  | Includes ZB1 |
| Bacteroidetes | Bacteroidetes | Bacteroidetes | Bacteroidetes |  |
| Bacteroidetes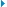Chlorobia | Chlorobi | Chlorobi |  |  |
| Bacteroidetes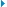OC31 | OC31 |  |  |  |
| Bacteroidetes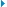Rhodothermia |  |  | Rhodothermaeota |  |
| Bacteroidetes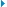Rhodothermia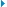Balneolales |  |  | Balneolaeota |  |
| BHI80-139 | BHI80-139 |  | Candidate division BHI80-139 |  |
| BRC1 | BRC1 | BRC1 |  |  |
| Caldiserica | Caldiserica | Caldiserica | Caldiserica | OP5, WCHB1-03 |
| Calditrichaeota | Caldithrix |  | Calditrichaeota | KSB1 |
| Calescamantes |  | Candidatus Calescamantes | Candidatus Calescamantes | EM19 |
| Chlamydiae | Chlamydiae | Chlamydiae | Chlamydiae | Includes GN13 |
| Chloroflexi | Chloroflexi | Chloroflexi | Chloroflexi | Includes MSBL5 |
| Chloroflexi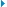AD3 | AD3 |  | Candidate division AD3 |  |
| Chloroflexi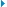Ktedonobacteria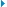MVP-21 | MVP-21 |  |  | GN14 |
| Chrysiogenetes | Chrysiogenetes | Chrysiogenetes | Chrysiogenetes |  |
| CK-2C2-2 |  |  |  |  |
| Cloacimonetes | WWE1 | Cloacimonetes | Candidatus Cloacimonetes | WWE1, MSBL2 |
| Coprothermobacterota |  |  | Coprothermobacterota |  |
| Cyanobacteria | Cyanobacteria | Cyanobacteria | Cyanobacteria | Oxyphotobacteria |
| Cyanobacteria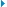Melainabacteria |  |  | Candidatus Melainabacteria |  |
| Dadabacteria | SBR1093 |  | Candidatus Dadabacteria | SBR1093 |
| Deferribacteres | Deferribacteres | Deferribacteres | Deferribacteres |  |
| Deinococcus-Thermus | Thermi | Deinococcus-Thermus | Deinococcus-Thermus |  |
| Dependentiae | TM6 |  | Candidatus Dependentiae | TM6 |
| Desantisbacteria |  |  | Candidatus Desantisbacteria |  |
| Dictyoglomi | Dictyoglomi | Dictyoglomi | Dictyoglomi |  |
| Edwardsbacteria |  |  | Candidatus Edwardsbacteria |  |
| Elusimicrobia | Elusimicrobia | Elusimicrobia | Elusimicrobia | TG1 |
| Entotheonellaeota |  |  | Candidatus Tectomicrobia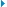Candidatus Entotheonella | Tectomicrobia |
| Epsilonbacteraeota |  |  |  |  |
| FBP | FBP |  | Abditibacteriota |  |
| FCPU426 | FCPU426 |  | Candidate division FCPU426 |  |
| Fervidibacteria | OctSpa1-106 |  | Candidatus Fervidibacteria | OctSpa1-106 |
| Fibrobacteres | Fibrobacteres | Fibrobacteres | Fibrobacteres | Includes GN09 and GOUTA4 |
| Fibrobacteres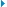Fibrobacteria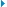Fibrobacterales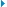Candidatus Raymondbacteria |  |  | Candidatus Raymondbacteria |  |
| Firestonebacteria |  |  | Candidatus Firestonebacteria |  |
| Firmicutes | Firmicutes | Firmicutes | Firmicutes | Bacillota |
| Fusobacteria | Fusobacteria | Fusobacteria | Fusobacteria |  |
| GAL15 | GAL15 |  | Candidate division GAL15 | Galena 15 |
| GBS-1 |  |  |  |  |
| Gemmatimonadetes | Gemmatimonadetes | Gemmatimonadetes | Gemmatimonadetes | KS-B, GN12 |
| GN01 | GN01 |  | Candidate division GN01 |  |
| Halanaerobiaeota |  |  | Firmicutes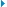Clostridia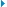Halanaerobiales |  |
| Hydrogenedentes | NKB19 | Hydrogenedentes | Candidatus Hydrogenedentes | NKB19 |
| Hydrothermae |  |  | Candidatus Hydrothermae | EM3 |
| Kiritimatiellaeota |  |  | Kiritimatiellaeota | Verrucomicrobia subdivision 5, MSBL3 |
| Latescibacteria | WS3 | Latescibacteria | Candidatus Latescibacteria | WS3, Eisenbacteria, GN03 |
| LCP-89 | LCP-89 |  |  |  |
| Lentisphaerae | Lentisphaerae | Lentisphaerae | Lentisphaerae | vadinBE97 |
| Lindowbacteria |  |  | Candidatus Lindowbacteria |  |
| Margulisbacteria |  |  | Candidatus Margulisbacteria |  |
| Marinimicrobia (SAR406 clade) | SAR406 | Marinimicrobia | Candidatus Marinimicrobia | Marine group A |
| MAT-CR-M4-B07 | MAT-CR-M4-B07 |  |  |  |
| Modulibacteria | KSB3 |  |  | KSB3, GN06 |
| Nitrospinae |  | Nitrospinae | Nitrospinae |  |
| Nitrospirae | Nitrospirae | Nitrospirae | Nitrospirae | Thermodesulfovibrio |
| Omnitrophicaeota | OP3 | Omnitrophica | Candidatus Omnitrophica | OP3, MSBL4 and includes WOR-2 |
| Patescibacteria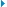ABY1 |  |  |  |  |
| Patescibacteria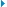ABY1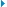Candidatus Buchananbacteria |  |  | Candidatus Buchananbacteria |  |
| Patescibacteria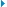ABY1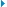Candidatus Falkowbacteria |  |  | Candidatus Falkowbacteria |  |
| Patescibacteria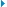ABY1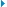Candidatus Jacksonbacteria |  |  | Candidatus Jacksonbacteria |  |
| Patescibacteria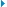ABY1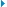Candidatus Kerfeldbacteria |  |  | Candidatus Kerfeldbacteria | Veblenbacteria |
| Patescibacteria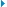ABY1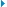Candidatus Komeilibacteria |  |  | Candidatus Komeilibacteria |  |
| Patescibacteria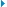ABY1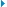Candidatus Kuenenbacteria |  |  | Candidatus Kuenenbacteria |  |
| Patescibacteria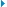ABY1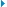Candidatus Magasanikbacteria |  |  | Candidatus Magasanikbacteria |  |
| Patescibacteria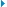ABY1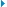Candidatus Uhrbacteria |  |  | Candidatus Uhrbacteria |  |
| Patescibacteria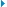Berkelbacteria |  |  | Candidatus Berkelbacteria | ACD58 |
| Patescibacteria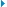CPR2 |  |  | Candidate division CPR2 |  |
| Patescibacteria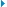Gracilibacteria |  |  | Candidatus Gracilibacteria | Includes GN02, GN07, GN08, BD1-5, ACD80 |
| Patescibacteria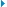Gracilibacteria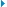Absconditabacteriales (SR1) | SR1 | SR1 | Candidate division SR1 | Absconditabacteria |
| Patescibacteria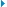Gracilibacteria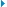Candidatus Abawacabacteria |  |  | Candidatus Abawacabacteria | GN10 |
| Patescibacteria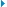Gracilibacteria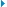Candidatus Peregrinibacteria |  |  | Candidatus Peregrinibacteria | PER, includes GN11 |
| Patescibacteria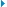Kazania | Kazan-3B-28 |  | Candidate division Kazan-3B-28 |  |
| Patescibacteria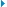MD2896-B216 |  |  |  |  |
| Patescibacteria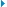Microgenomatia | OP11 | Microgenomates | Candidatus Microgenomates | CPR3 |
| Patescibacteria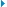Microgenomatia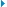Candidatus Amesbacteria |  |  | Candidatus Amesbacteria |  |
| Patescibacteria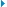Microgenomatia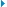Candidatus Beckwithbacteria |  |  | Candidatus Beckwithbacteria |  |
| Patescibacteria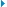Microgenomatia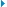Candidatus Chrisholmbacteria |  |  | Candidatus Chisholmbacteria |  |
| Patescibacteria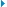Microgenomatia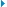Candidatus Collierbacteria |  |  | Candidatus Collierbacteria |  |
| Patescibacteria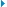Microgenomatia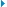Candidatus Curtissbacteria |  |  | Candidatus Curtissbacteria |  |
| Patescibacteria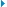Microgenomatia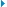Candidatus Daviesbacteria |  |  | Candidatus Daviesbacteria |  |
| Patescibacteria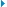Microgenomatia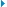Candidatus Gottesmanbacteria |  |  | Candidatus Gottesmanbacteria |  |
| Patescibacteria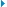Microgenomatia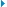Candidatus Levybacteria |  |  | Candidatus Levybacteria |  |
| Patescibacteria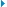Microgenomatia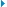Candidatus Pacebacteria |  |  | Candidatus Pacebacteria |  |
| Patescibacteria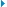Microgenomatia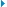Candidatus Roizmanbacteria |  |  | Candidatus Roizmanbacteria |  |
| Patescibacteria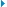Microgenomatia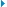Candidatus Shapirobacteria |  |  | Candidatus Shapirobacteria |  |
| Patescibacteria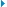Microgenomatia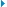Candidatus Woesebacteria |  |  | Candidatus Woesebacteria | Blackburnbacteria |
| Patescibacteria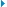Microgenomatia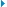Candidatus Woykebacteria |  |  | Candidatus Woykebacteria |  |
| Patescibacteria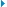Parcubacteria | OD1 | Parcubacteria | Candidatus Parcubacteria |  |
| Patescibacteria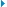Parcubacteria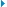Candidatus Adlerbacteria |  |  | Candidatus Adlerbacteria |  |
| Patescibacteria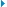Parcubacteria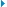Candidatus Azambacteria |  |  | Candidatus Azambacteria |  |
| Patescibacteria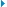Parcubacteria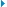Candidatus Brennerbacteria |  |  | Candidatus Brennerbacteria |  |
| Patescibacteria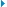Parcubacteria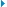Candidatus Campbellbacteria |  |  | Candidatus Campbellbacteria |  |
| Patescibacteria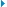Parcubacteria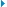Candidatus Colwellbacteria |  |  | Candidatus Colwellbacteria |  |
| Patescibacteria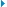Parcubacteria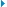Candidatus Doudnabacteria |  |  | Candidatus Doudnabacteria | SM2F11 |
| Patescibacteria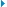Parcubacteria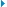Candidatus Giovannonibacteria |  |  | Candidatus Giovannonibacteria |  |
| Patescibacteria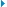Parcubacteria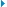Candidatus Jorgensenbacteria |  |  | Candidatus Jorgensenbacteria |  |
| Patescibacteria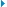Parcubacteria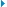Candidatus Kaiserbacteria |  |  | Candidatus Kaiserbacteria |  |
| Patescibacteria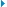Parcubacteria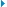Candidatus Liptonbacteria |  |  | Candidatus Liptonbacteria |  |
| Patescibacteria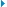Parcubacteria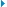Candidatus Lloydbacteria |  |  | Candidatus Lloydbacteria |  |
| PatescibacteriaParcubacteriaCandidatus Moranbacteria |  |  | Candidatus Moranbacteria |  |
| PatescibacteriaParcubacteriaCandidatus Nealsonbacteria |  |  | Candidatus Nealsonbacteria |  |
| PatescibacteriaParcubacteriaCandidatus Nomurabacteria |  |  | Candidatus Nomurabacteria |  |
| PatescibacteriaParcubacteriaCandidatus Portnoybacteria |  |  | Candidatus Portnoybacteria |  |
| PatescibacteriaParcubacteriaCandidatus Ryanbacteria |  |  | Candidatus Ryanbacteria |  |
| PatescibacteriaParcubacteriaCandidatus Spechtbacteria |  |  | Candidatus Spechtbacteria |  |
| PatescibacteriaParcubacteriaCandidatus Staskawiczbacteria |  |  | Candidatus Staskawiczbacteria |  |
| PatescibacteriaParcubacteriaCandidatus Terrybacteria |  |  | Candidatus Terrybacteria |  |
| PatescibacteriaParcubacteriaCandidatus Vogelbacteria |  |  | Candidatus Vogelbacteria |  |
| PatescibacteriaParcubacteriaCandidatus Wolfebacteria |  |  | Candidatus Wolfebacteria |  |
| PatescibacteriaParcubacteriaCandidatus Yanofskybacteria |  |  | Candidatus Yanofskybacteria |  |
| PatescibacteriaParcubacteriaCandidatus Yonathbacteria |  |  | Candidatus Yonathbacteria |  |
| PatescibacteriaParcubacteriaCandidatus Zambryskibacteria |  |  | Candidatus Zambryskibacteria |  |
| PatescibacteriaParcubacteriaGWA2-38-13b |  |  | Candidatus Andersenbacteria | Andersenbacteria |
| PatescibacteriaSaccharimonadia | TM7 | Candidatus Saccharibacteria | Candidatus Saccharibacteria | TM7 |
| PatescibacteriaWS6 (Dojkabacteria) | WS6 |  | Candidatus Dojkabacteria | Candidate division WS6 |
| PatescibacteriaWWE3 |  |  | Candidate division WWE3 | Katanobacteria |
| PAUC34f | PAUC34f |  |  |  |
| Planctomycetes | Planctomycetes | Planctomycetes | Planctomycetes |  |
| Poribacteria | Poribacteria | Poribacteria | Candidatus Poribacteria |  |
| Proteobacteria | Proteobacteria | Proteobacteria | Proteobacteria |  |
| ProteobacteriaDeltaproteobacteriaThermodesulfobacteriales |  | Thermodesulfobacteria | Thermodesulfobacteria |  |
| Rokubacteria |  |  | Candidatus Rokubacteria | CSP1-6, Candidate division SPAM |
| RokubacteriaNC10 | NC10 |  | Candidate division NC10 |  |
| RsaHF231 |  |  |  |  |
| Schekmanbacteria |  |  | Candidatus Schekmanbacteria |  |
| Spirochaetes | Spirochaetes | Spirochaetes | Spirochaetes | Includes GN05 |
| Synergistetes | Synergistetes | Synergistetes | Synergistetes |  |
| TA06 | TA06 |  | Candidate division TA06 |  |
| Tenericutes | Tenericutes | Tenericutes | Tenericutes |  |
| Thermosulfidibacteraeota |  |  | AquificaeAquificalesThermosulfidibacter |  |
| Thermotogae | Thermotogae | Thermotogae | Thermotogae |  |
| Verrucomicrobia | Verrucomicrobia |  | Verrucomicrobia |  |
| WOR-1 |  |  |  |  |
| WPS-2 | WPS-2 | Candidate division WPS-2 | Candidate division WPS-2 |  |
| WS1 | WS1 |  | Candidate division WS1 |  |
| WS2 | WS2 |  | Candidate division WS2 |  |
| WS4 | WS4 |  | Candidate division WS4 |  |
| Zixibacteria |  |  | Candidate division Zixibacteria | RBG-1, GN04, GN15 |
|  | AC1 |  | Candidate division AC1 |  |
|  |  |  | Candidate division CAB-I |  |
|  |  |  | Candidate division JL-ETNP-Z39 |  |
|  |  |  | Candidate division KD3-62 |  |
|  |  |  | Candidate division kpj58rc |  |
|  |  |  | Candidate division KSA1 |  |
|  |  |  | Candidate division KSA2 |  |
|  |  |  | Candidate division KSB2 |  |
|  |  |  | Candidate division KSB4 |  |
| Polyphyletic | NPL-UPA2 |  | Candidate division NPL-UPA2 |  |
|  |  |  | Candidate division NT-B4 |  |
|  |  |  | Candidate division OP2 |  |
|  |  |  | Candidate division OP4 |  |
|  |  |  | Candidate division OP6 |  |
|  |  |  | Candidate division OP7 |  |
|  |  |  | Candidate division OS-K |  |
|  |  |  | Candidate division RF3 |  |
|  |  |  | Candidate division SAM |  |
|  |  |  | Candidate division Sediment-1 | |
|  |  |  | Candidate division Sediment-2 | |
|  |  |  | Candidate division Sediment-3 | |
|  |  |  | Candidate division Sediment-4 | |
|  |  |  | Candidate division TG2 |  |
|  |  |  | Candidate division VC2 |  |
|  |  |  | Candidate division WOR-3 |  |
|  |  | Candidate division WPS-1 | Candidate division WPS-1 |  |
|  | WS5 |  | Candidate division WS5 |  |
|  |  |  | Candidate division WYO |  |
|  | ZB3 | Candidate division ZB3 | Candidatus MargulisbacteriaCandidatus Marinamargulisbacteria |  |
|  |  |  | Candidatus Abyssubacteria | SURF-CP-1 |
|  |  |  | Candidatus Aureabacteria |  |
|  |  |  | Candidatus Coatesbacteria |  |
|  |  |  | Candidatus Delongbacteria |  |
|  |  |  | Candidatus Fermentibacteria |  |
|  |  |  | Candidatus Glassbacteria |  |
|  |  |  | Candidatus Goldbacteria |  |
|  |  |  | Candidatus Handelsmanbacteria |  |
| Polyphyletic |  |  | Candidatus Niyogibacteria |  |
|  |  |  | Candidatus Riflebacteria |  |
|  |  |  | Candidatus Sumerlaeota |  |
| Polyphyletic |  |  | Candidatus Sungbacteria |  |
|  |  |  | Candidatus Tagabacteria |  |
| Polyphyletic |  |  | Candidatus Taylorbacteria |  |
| Polyphyletic |  |  | Candidatus Wildermuthbacteria |  |
|  |  |  | Candidate division CPR1 |  |
|  | H-178 |  |  |  |
|  |  |  | Candidatus Kryptonia |  |
|  | LDI |  |  |  |
|  | MVS-104 |  |  |  |
|  | SC4 |  |  |  |
|  | VHS-B3-43 |  |  |  |
|  |  |  | Candidatus Wallbacteria |  |
|  |  |  | Candidatus Wirthbacteria |  |

^a^ The following phylum-level groups have been proposed but the names are not listed in any of the four databases: Guyamas1 (Teske et al., 2002) and SC3 (Dunbar et al., 2002).

^b^ References for databases used: SILVA 132 (Quast et al., 2013;Yilmaz et al., 2014), RDP (Cole et al., 2014), GreenGenes (DeSantis et al., 2006), NCBI Taxonomy Browser (Benson et al., 2009;Sayers et al., 2009).

**References**

Benson, D.A., Karsch-Mizrachi, I., Lipman, D.J., Ostell, J., and Sayers, E.W. (2009). GenBank. *Nucleic Acids Research* 37**,** D26-D31.

Cole, J.R., Wang, Q., Fish, J.A., Chai, B.L., Mcgarrell, D.M., Sun, Y.N., Brown, C.T., Porras-Alfaro, A., Kuske, C.R., and Tiedje, J.M. (2014). Ribosomal Database Project: data and tools for high throughput rRNA analysis. *Nucleic Acids Research* 42**,** D633-D642.

Desantis, T.Z., Hugenholtz, P., Larsen, N., Rojas, M., Brodie, E.L., Keller, K., Huber, T., Dalevi, D., Hu, P., and Andersen, G.L. (2006). Greengenes, a chimera-checked 16S rRNA gene database and workbench compatible with ARB. *Applied and Environmental Microbiology* 72**,** 5069-5072.

Dunbar, J., Barns, S.M., Ticknor, L.O., and Kuske, C.R. (2002). Empirical and Theoretical Bacterial Diversity in Four Arizona Soils. *Applied and Environmental Microbiology* 68**,** 3035-3045.

Quast, C., Pruesse, E., Yilmaz, P., Gerken, J., Schweer, T., Yarza, P., Peplies, J., and Glockner, F.O. (2013). The SILVA ribosomal RNA gene database project: improved data processing and web-based tools. *Nucleic Acids Research* 41**,** D590-D596.

Sayers, E.W., Barrett, T., Benson, D.A., Bryant, S.H., Canese, K., Chetvernin, V., Church, D.M., Dicuccio, M., Edgar, R., Federhen, S., Feolo, M., Geer, L.Y., Helmberg, W., Kapustin, Y., Landsman, D., Lipman, D.J., Madden, T.L., Maglott, D.R., Miller, V., Mizrachi, I., Ostell, J., Pruitt, K.D., Schuler, G.D., Sequeira, E., Sherry, S.T., Shumway, M., Sirotkin, K., Souvorov, A., Starchenko, G., Tatusova, T.A., Wagner, L., Yaschenko, E., and Ye, J. (2009). Database resources of the National Center for Biotechnology Information. *Nucleic Acids Research* 37**,** 3124-3124.

Teske, A., Hinrichs, K.-U., Edgcomb, V., De Vera Gomez, A., Kysela, D., Sylva, S.P., Sogin, M.L., and Jannasch, H.W. (2002). Microbial Diversity of Hydrothermal Sediments in the Guaymas Basin: Evidence for Anaerobic Methanotrophic Communities. *Applied and Environmental Microbiology* 68**,** 1994-2007.

Yilmaz, P., Parfrey, L.W., Yarza, P., Gerken, J., Pruesse, E., Quast, C., Schweer, T., Peplies, J., Ludwig, W., and Glockner, F.O. (2014). The SILVA and "All-species Living Tree Project (LTP)" taxonomic frameworks. *Nucleic Acids Research* 42**,** D643-D648.
