# Supplementary material for: Uncovering the Uncultivated Majority in Antarctic Soils: Toward a Synergistic Approach
Source: Front Microbiol. 2019 Feb 15;10:242. doi: 10.3389/fmicb.2019.00242 (PMC6385771; doi:10.3389/fmicb.2019.00242)
Supplement: Table S2 — List of valid prokaryotic species that have been described with a type strain originating from Antarctica, listed alphabetically per phylum. Genera first described from Antarctica are marked with a ∗. Genera recovered during isolation campaigns involving Antarctic soil are marked with a †. [file Table_2.DOCX]

Table S2. List of valid prokaryotic species that have been described with a type strain originating from Antarctica, listed alphabetically per phylum. Genera first described from Antarctica are marked with a *. Genera recovered during isolation campaigns involving Antarctic soil are marked with a †.

| **Species** | |  | |  | | **Reference(s)** |
| --- | --- | --- | --- | --- | --- | --- |
| **Archaea** | |  | |  | |  |
| **Euryarchaeota** | |  | |  | |  |
| * | *Halohasta* | | | *litchfieldiae* | | (Mou et al., 2012) |
| * | *Halorubrum* | | | *lacusprofundi* | | (Franzmann et al., 1988) |
| * | *Methanococcoides* | | | *burtonii* | | (Franzmann et al., 1992) |
| * | *Methanogenium* | | | *frigidum* | | (Franzmann et al., 1997) |
| **Bacteria** | |  | |  | |  |
| **Abditibacteriota** | |  | |  | |  |
| *† | *Abditibacterium* | | | *utseinense* | | (Tahon et al., 2018) |
| **Actinobacteria** | |  | |  | |  |
|  | *Amycolatopsis* | | | *antarctica* | | (Wang et al., 2018) |
| † | *Arthrobacter* | | | *antarcticus* | | (Pindi et al., 2010) |
|  |  | | | *ardleyensis* | | (Chen et al., 2005) |
|  |  | | | *cryotolerans* | | (Ganzert et al., 2011a) |
|  |  | | | *flavus* | | (Reddy et al., 2000) |
|  |  | | | *gangotriensis* | | (Gupta et al., 2004) |
|  |  | | | *kerguelensis* | | (Gupta et al., 2004) |
|  |  | | | *livingstonensis* | | (Ganzert et al., 2011a) |
|  |  | | | *psychrochitiniphilus* | | (Wang et al., 2009) |
|  |  | | | *roseus* | | (Reddy et al., 2002) |
| *† | *Friedmanniella* | | | *antarctica* | | (Schumann et al., 1997) |
|  |  | | | *lacustris* | | (Lawson et al., 2000) |
| † | *Kocuria* | | | *polaris* | | (Reddy et al., 2003b) |
| † | *Leifsonia* | | | *antarctica* | | (Pindi et al., 2009) |
|  |  | | | *aurea* | | (Reddy et al., 2003c) |
|  |  | | | *kafniensis* | | (Pindi et al., 2009) |
|  |  | | | *psychrotolerans* | | (Ganzert et al., 2011b) |
|  |  | | | *rubra* | | (Reddy et al., 2003c) |
| * | *Marisediminicola* | | | *antarctica* | | (Li et al., 2010) |
| † | *Micrococcus* | | | *antarcticus* | | (Liu et al., 2000) |
| † | *Micromonospora* | | | *endolithica* | | (Hirsch et al., 2004b) |
| *† | *Modestobacter* | | | *multiseptatus* | | (Mevs et al., 2000) |
| † | *Nesterenkonia* | | | *aurantiaca* | | (Finore et al., 2016) |
|  |  | | | *lacusekhoensis* | | (Collins et al., 2002) |
| † | *Nocardioides* | | | *aquaticus* | | (Lawson et al., 2000) |
| † | *Pseudonocardia* | | | *antarctica* | | (Prabahar et al., 2004) |
| *† | *Raineyella* | | | *antarctica* | | (Pikuta et al., 2016) |
| † | *Rhodococcus* | | | *psychrotolerans* | | (Silva et al., 2018) |
| * | *Rhodoglobus* | | | *vestalii* | | (Sheridan et al., 2003) |
|  | *Sanguibacter* | | | *antarcticus* | | (Hong et al., 2008) |
|  |  | | | *gelidistatuariae* | | (Pikuta et al., 2017b) |
| † | *Streptomyces* | | | *fildesensis* | | (Li et al., 2011b) |
|  |  | | | *hypolithicus* | | (Le Roes-Hill et al., 2009) |
| **Bacteroidetes** | |  | |  | |  |
|  | *Aequorivita* | | | *viscosa* | | (Liu et al., 2013) |
|  | *Algoriphagus* | | | *antarcticus* | | (Van Trappen et al., 2004a) |
| * | *Antarcticibacterium* | | | *flavum* | | (Li et al., 2018b) |
| * | *Antarcticimonas* | | | *flava* | | (Yang et al., 2009) |
| * | *Aquaticitalea* | | | *lipolytica* | | (Xarnxidin et al., 2016) |
|  | *Arenibacter* | | | *antarcticus* | | (Li et al., 2017) |
|  | *Bizionia* | | | *algoritergicola* | | (Bowman and Nichols, 2005) |
|  |  | | | *argentinensis* | | (Bercovich et al., 2008) |
|  |  | | | *gelidisalsuginis* | | (Bowman and Nichols, 2005) |
|  |  | | | *myxarmorum* | | (Bowman and Nichols, 2005) |
|  |  | | | *saleffrena* | | (Bowman and Nichols, 2005) |
|  | *Cellulophaga* | | | *algicola* | | (Bowman, 2000) |
| * | *Changchengzhania* | | | *lutea* | | (Wang et al., 2017) |
| † | *Flavitalea* | | | *antarctica* | | (Wei et al., 2017) |
| † | *Flavobacterium* | | | *antarcticum* | | (Yi et al., 2005a) |
|  |  | | | *ardleyense* | | (Zhou et al., 2017) |
|  |  | | | *chryseum* | | (Kralova et al., 2018) |
|  |  | | | *collinsense* | | (Zhang et al., 2016) |
|  |  | | | *degerlachei* | | (Van Trappen et al., 2004c) |
|  |  | | | *faecale* | | (Kim et al., 2014) |
|  |  | | | *frigidarium* | | (Humphry et al., 2001) |
|  |  | | | *frigidimaris* | | (Nogi et al., 2005) |
|  |  | | | *frigoris* | | (Van Trappen et al., 2004c) |
|  |  | | | *fryxellicola* | | (Van Trappen et al., 2005) |
|  |  | | | *gelidilacus* | | (Van Trappen et al., 2003) |
|  |  | | | *gillisiae* | | (McCammon and Bowman, 2000) |
|  |  | | | *gondwanense* | | (Dobson et al., 1993) |
|  |  | | | *hibernum* | | (McCammon et al., 1998) |
|  |  | | | *kingsejongi* | | (Choi et al., 2018) |
|  |  | | | *micromati* | | (Van Trappen et al., 2004c) |
|  |  | | | *ovatum* | | (Ren et al., 2018) |
|  |  | | | *phocarum* | | (Zhou et al., 2018) |
|  |  | | | *psychrolimnae* | | (Van Trappen et al., 2005) |
|  |  | | | *psychroterrae* | | (Kralova et al., 2018) |
|  |  | | | *salegens* | | (Dobson et al., 1993) |
|  |  | | | *segetis* | | (Yi and Chun, 2006) |
|  |  | | | *tegetincola* | | (McCammon and Bowman, 2000) |
|  |  | | | *weaverense* | | (Yi and Chun, 2006) |
|  |  | | | *xanthum* | | (McCammon and Bowman, 2000) |
|  | *Flectobacillus* | | | *glomeratus* | | (McGuire et al., 1987) |
| * | *Gelidibacter* | | | *algens* | | (Bowman et al., 1997a) |
|  |  | | | *gilvus* | | (Bowman and Nichols, 2005) |
|  |  | | | *salicanalis* | | (Bowman and Nichols, 2005) |
| * | *Gillisia* | | | *hiemivivida* | | (Bowman and Nichols, 2005) |
|  |  | | | *illustrilutea* | | (Bowman and Nichols, 2005) |
|  |  | | | *limnaea* | | (Van Trappen et al., 2004d) |
|  |  | | | *sandarakina* | | (Bowman and Nichols, 2005) |
|  | *Gramella* | | | *antarctica* | | (Li et al., 2018a) |
| *† | *Hymenobacter* | | | *coccineus* | | (Sedlacek et al., 2017a) |
|  |  | | | *glacialis* | | (Sedlacek et al., 2017a) |
|  |  | | | *lapidarius* | | (Sedlacek et al., 2017a) |
|  |  | | | *nivis* | | (Kojima et al., 2016) |
|  |  | | | *roseosalivarius* | | (Hirsch et al., 1998) |
|  |  | | | *rubripertinctus* | | (Jiang et al., 2018) |
|  | *Kordia* | | | *antarctica* | | (Baek et al., 2013) |
| * | *Lacinutrix* | | | *copepodicola* | | (Bowman and Nichols, 2005) |
|  |  | | | *jangbogonensis* | | (Lee et al., 2014) |
|  | *Lewinella* | | | *antarctica* | | (Oh et al., 2009) |
|  | *Maribacter* | | | *antarcticus* | | (Zhang et al., 2009) |
| † | *Mucilaginibacter* | | | *antarcticus* | | (Zheng et al., 2016) |
|  |  | | | *terrae* | | (Sedlacek et al., 2017b) |
|  | *Muricauda* | | | *antarctica* | | (Wu et al., 2013) |
|  | *Nonlabens* | | | *antarcticus* | | (Kwon et al., 2014) |
| † | *Pedobacter* | | | *ardleyensis* | | (Da et al., 2015) |
|  |  | | | *jamesrossensis* | | (Švec et al., 2017) |
|  |  | | | *lithocola* | | (Švec et al., 2017) |
|  |  | | | *mendelii* | | (Švec et al., 2017) |
|  |  | | | *petrophilus* | | (Švec et al., 2017) |
|  | *Polaribacter* | | | *sejongensis* | | (Kim et al., 2013) |
| * | *Pricia* | | | *antarctica* | | (Yu et al., 2012) |
| * | *Psychroflexus* | | | *lacisalsi* | | (Zhang et al., 2010a) |
|  |  | | | *torquis* | | (Bowman et al., 1998c) |
| * | *Psychroserpens* | | | *burtonensis* | | (Bowman et al., 1997a) |
|  |  | | | *jangbogonensis* | | (Baek et al., 2015) |
| *† | *Sejongia* | | | *antarctica* | | (Yi et al., 2005b) |
|  |  | | | *jeonii* | | (Yi et al., 2005b) |
|  |  | | | *marina* | | (Lee et al., 2007c) |
| † | *Sphingobacterium* | | | *antarcticus* | | (Shivaji et al., 1992) |
| * | *Subsaxibacter* | | | *broadyi* | | (Bowman and Nichols, 2005) |
| * | *Subsaximicrobium* | | | *saxinquilinus* | | (Bowman and Nichols, 2005) |
|  |  | | | *wynnwilliamsii* | | (Bowman and Nichols, 2005) |
|  | *Ulvibacter* | | | *antarcticus* | | (Choi et al., 2007) |
| * | *Williamwhitmania* | | | *taraxaci* | | (Pikuta et al., 2017a) |
| **Cyanobacteria** | |  | |  | |  |
| * | *Aliterella* | | | *atlantica* | | (Rigonato et al., 2016) |
|  |  | | | *antarctica* | | (Rigonato et al., 2016) |
| **Deinococcus-Thermus** | | |  | |  |  |
| † | *Deinococcus* | | | *antarcticus* | | (Dong et al., 2015) |
|  |  | | | *frigens* | | (Hirsch et al., 2004a) |
|  |  | | | *marmoris* | | (Hirsch et al., 2004a) |
|  |  | | | *rubellus* | | (Choi et al., 2016) |
|  |  | | | *rubrus* | | (Srinivasan et al., 2017) |
|  |  | | | *saxicola* | | (Hirsch et al., 2004a) |
| **Firmicutes** | |  | |  | |  |
| † | *Alicyclobacillus* | | | *pohliae* | | (Imperio et al., 2008) |
| † | *Aneurinibacillus* | | | *terranovensis* | | (Allan et al., 2005) |
| † | *Anoxybacillus* | | | *amylolyticus* | | (Poli et al., 2006) |
| † | *Bacillus* | | | *fumarioli* | | (Logan et al., 2000) |
|  |  | | | *piscis* | | (Lee et al., 2016) |
|  |  | | | *thermoantarcticus* | | (Nicolaus et al., 1996) |
| † | *Brevibacillus* | | | *levickii* | | (Allan et al., 2005) |
| † | *Carnobacterium* | | | *alterfunditum* | | (Franzmann et al., 1991) |
|  |  | | | *antarcticum* | | (Zhu et al., 2018) |
|  |  | | | *funditum* | | (Franzmann et al., 1991) |
|  |  | | | *iners* | | (Snauwaert et al., 2013) |
|  | *Clostridium* | | | *bowmanii* | | (Spring et al., 2003) |
|  |  | | | *frigoris* | | (Spring et al., 2003) |
|  |  | | | *lacusfryxellense* | | (Spring et al., 2003) |
|  |  | | | *psychrophilum* | | (Spring et al., 2003) |
|  |  | | | *schirmacherense* | | (Alam et al., 2006) |
|  |  | | | *vincentii* | | (Mountfort et al., 1997) |
| † | *Exiguobacterium* | | | *antarcticum* | | (Fruhling et al., 2002) |
|  |  | | | *soli* | | (Chaturvedi et al., 2008) |
| † | *Paenibacillus* | | | *antarcticus* | | (Montes et al., 2004) |
|  |  | | | *cineris* | | (Logan et al., 2004) |
|  |  | | | *cookii* | | (Logan et al., 2004) |
|  |  | | | *wynnii* | | (Rodriguez-Diaz et al., 2005) |
| † | *Planococcus* | | | *faecalis* | | (Kim et al., 2015) |
|  |  | | | *maitriensis* | | (Alam et al., 2003) |
|  |  | | | *mcmeekinii* | | (Junge et al., 1998) |
| * | *Psychrosinus* | | | *fermentans* | | (Sattley et al., 2008) |
| † | *Sporosarcina* | | | *antarctica* | | (Yu et al., 2008) |
|  |  | | | *macmurdoensis* | | (Reddy et al., 2003a) |
| † | *Staphylococcus* | | | *edaphicus* | | (Pantucek et al., 2018) |
| **Proteobacteria** | |  | |  | |  |
| * | *Actimicrobium* | | | *antarcticum* | | (Kim et al., 2011) |
| * | *Allohahella* | | | *marinimesophila* | | (Han et al., 2016) |
|  | *Alteromonas* | | | *stellipolaris* | | (Van Trappen et al., 2004b) |
|  | *Campylobacter* | | | *subantarcticus* | | (Debruyne et al., 2010) |
|  | *Colwellia* | | | *demingiae* | | (Bowman et al., 1998a) |
|  |  | | | *hornerae* | | (Bowman et al., 1998a) |
|  |  | | | *psychrotropica* | | (Bowman et al., 1998a) |
|  |  | | | *rossensis* | | (Bowman et al., 1998a) |
| *† | *Constrictibacter* | | | *antarcticus* | | (Yamada et al., 2011) |
|  | *Desulfovibrio* | | | *lacusfryxellense* | | (Sattley and Madigan, 2010) |
| * | *Glaciecola* | | | *pallidula* | | (Bowman et al., 1998b) |
|  |  | | | *punicea* | | (Bowman et al., 1998b) |
| * | *Granulosicoccus* | | | *antarcticus* | | (Lee et al., 2007a) |
|  | *Hahella* | | | *antarctica* | | (Lee et al., 2008) |
|  | *Halomonas* | | | *alkaliantarctica* | | (Poli et al., 2007) |
|  |  | | | *glaciei* | | (Reddy et al., 2003d) |
|  | *Kiloniella* | | | *antarctica* | | (Si et al., 2017) |
| * | *Loktanella* | | | *fryxellensis* | | (Van Trappen et al., 2004a) |
|  |  | | | *salsilacus* | | (Van Trappen et al., 2004a) |
|  |  | | | *vestfoldensis* | | (Van Trappen et al., 2004a) |
| † | *Lysobacter* | | | *oligotrophicus* | | (Fukuda et al., 2013) |
|  | *Marinobacter* | | | *antarcticus* | | (Liu et al., 2012) |
|  |  | | | *gelidimuriae* | | (Chua et al., 2018) |
|  |  | | | *guineae* | | (Montes et al., 2008) |
|  | *Marinomonas* | | | *ushuaiensis* | | (Prabagaran et al., 2005) |
| * | *Methylosphaera* | | | *hansonii* | | (Bowman et al., 1997c) |
|  | *Neptunomonas* | | | *antarctica* | | (Zhang et al., 2010b) |
|  | *Oceanicola* | | | *antarcticus* | | (Huo et al., 2014) |
| * | *Octadecabacter* | | | *antarcticus* | | (Gosink et al., 1997) |
| * | *Oleispira* | | | *antarctica* | | (Yakimov et al., 2003) |
|  | *Pararhizobium* | | | *antarcticum* | | (Naqvi et al., 2017) |
| *† | *Polaromonas* | | | *vacuolata* | | (Irgens et al., 1996) |
| *† | *Polymorphobacter* | | | *multimanifer* | | (Fukuda et al., 2014) |
|  | *Pseudoalteromonas* | | | *antarctica* | | (Bozal et al., 1997) |
|  |  | | | *neustonica* | | (Hwang et al., 2016) |
|  |  | | | *prydzensis* | | (Bowman, 1998) |
| † | *Pseudomonas* | | | *antarctica* | | (Reddy et al., 2004) |
|  |  | | | *deceptionensis* | | (Carrion et al., 2011) |
|  |  | | | *extremaustralis* | | (Lopez et al., 2009) |
|  |  | | | *gregormendelii* | | (Kosina et al., 2016) |
|  |  | | | *guineae* | | (Bozal et al., 2007) |
|  |  | | | *meridiana* | | (Reddy et al., 2004) |
|  |  | | | *pelagia* | | (Hwang et al., 2009) |
|  |  | | | *prosekii* | | (Kosina et al., 2013) |
|  |  | | | *proteolytica* | | (Reddy et al., 2004) |
|  |  | | | *versuta* | | (See-Too et al., 2017) |
| * | *Pseudooceanicola* | | | *atlanticus* | | (Lai et al., 2015) |
|  | *Pseudorhodobacter* | | | *antarcticus* | | (Chen et al., 2013) |
|  | *Psychrobacter* | | | *adeliensis* | | (Shivaji et al., 2004) |
|  |  | | | *aquaticus* | | (Shivaji et al., 2005) |
|  |  | | | *fozii* | | (Bozal et al., 2003) |
|  |  | | | *glacincola* | | (Bowman et al., 1997d) |
|  |  | | | *luti* | | (Bozal et al., 2003) |
|  |  | | | *nivimaris* | | (Heuchert et al., 2004) |
|  |  | | | *proteolyticus* | | (Denner et al., 2001) |
|  |  | | | *salsus* | | (Shivaji et al., 2004) |
|  |  | | | *vallis* | | (Shivaji et al., 2005) |
| *† | *Psychromonas* | | | *antarcticus* | | (Mountfort et al., 1998) |
|  |  | | | *aquatilis* | | (Kampfer et al., 2017) |
| * | *Puniceibacterium* | | | *antarcticum* | | (Liu et al., 2014) |
| † | *Rhodoferax* | | | *antarcticus* | | (Madigan et al., 2000) |
| * | *Rhodoligotrophos* | | | *appendicifer* | | (Fukuda et al., 2012) |
| * | *Robiginftomaculum* | | | *antarcticum* | | (Lee et al., 2007b) |
| * | *Roseicitreum* | | | *antarcticum* | | (Yu et al., 2011) |
| * | *Roseisalinus* | | | *antarcticus* | | (Labrenz et al., 2005) |
|  | *Roseovarius* | | | *antarcticus* | | (Deng et al., 2015) |
| * | *Saccharospirillum* | | | *impatiens* | | (Labrenz et al., 2003) |
|  | *Shewanella* | | | *frigidimarina* | | (Bowman et al., 1997b; Bozal et al., 2002) |
|  |  | | | *gelidimarina* | | (Bowman et al., 1997b) |
|  |  | | | *livingstonensis* | | (Bozal et al., 2002) |
|  |  | | | *vesiculosa* | | (Lozal et al., 2009) |
| † | *Sphingomonas* | | | *aerolata* | | (Busse et al., 2003) |
|  |  | | | *antarctica* | | (Huang et al., 2017) |
|  |  | | | *aurantiaca* | | (Busse et al., 2003) |
|  |  | | | *faeni* | | (Busse et al., 2003) |
| * | *Staleya* | | | *guttiformis* | | (Labrenz et al., 2000) |
|  | *Sulfitobacter* | | | *brevis* | | (Labrenz et al., 2000) |
|  | *Thalassospira* | | | *lohafexi* | | (Shivaji et al., 2015) |
| * | *Zhongshania* | | | *antarctica* | | (Li et al., 2011a) |
|  |  | | | *guokunii* | | (Li et al., 2011a) |

**References**

Alam, S.I., Dixit, A., Reddy, G.S.N., Dube, S., Palit, M., Shivaji, S., and Singh, L. (2006). Clostridium schirmacherense sp nov., an obligately anaerobic, proteolytic, psychrophilic bacterium isolated from lake sediment of Schirmacher Oasis, Antarctica. *International Journal of Systematic and Evolutionary Microbiology* 56**,** 715-720.

Alam, S.I., Singh, L., Dube, S., Reddy, G.S.N., and Shivaji, S. (2003). Psychrophilic Planococcus maitriensis sp.nov from Antarctica. *Systematic and Applied Microbiology* 26**,** 505-510.

Allan, R.N., Lebbe, L., Heyrman, J., De Vos, P., Buchanan, C.J., and Logan, N.A. (2005). Brevibacillus levickii sp nov and Aneurinibacillus terranovensis sp nov., two novel thermoacidophiles isolated from geothermal soils of northern Victoria Land, Antarctica. *International Journal of Systematic and Evolutionary Microbiology* 55**,** 1039-1050.

Baek, K., Choi, A., Kang, I., Lee, K., and Cho, J.C. (2013). Kordia antarctica sp nov., isolated from Antarctic seawater. *International Journal of Systematic and Evolutionary Microbiology* 63**,** 3617-3622.

Baek, K., Lee, Y.M., Hwang, C.Y., Park, H., Jung, Y.J., Kim, M.K., Hong, S.G., Kim, J.H., and Lee, H.K. (2015). Psychroserpens jangbogonensis sp nov., a psychrophilic bacterium isolated from Antarctic marine sediment. *International Journal of Systematic and Evolutionary Microbiology* 65**,** 183-188.

Bercovich, A., Vazquez, S.C., Yankilevich, P., Coria, S.H., Foli, M., Hernandez, E., Vidal, A., Ruberto, L., Melo, C., Marenssi, S., Criscuolo, M., Memoli, M., Arguelles, M., and Mac Cormack, W.P. (2008). Bizionia argentinensis sp nov., isolated from surface marine water in Antarctica. *International Journal of Systematic and Evolutionary Microbiology* 58**,** 2363-2367.

Bowman, J.P. (1998). Pseudoalteromonas prydzensis sp. nov., a psychrotrophic, halotolerant bacterium from Antarctic sea ice. *International Journal of Systematic Bacteriology* 48**,** 1037-1041.

Bowman, J.P. (2000). Description of Cellulophaga algicola sp. nov., isolated from the surfaces of Antarctic algae, and reclassification of Cytophaga uliginosa (ZoBell and Upham 1944) Reichenbach 1989 as Cellulophaga uliginosa comb. nov. *International Journal of Systematic and Evolutionary Microbiology* 50**,** 1861-1868.

Bowman, J.P., Gosink, J.J., Mccammon, S.A., Lewis, T.E., Nichols, D.S., Nichols, P.D., Skerratt, J.H., Staley, J.T., and Mcmeekin, T.A. (1998a). Colwellia demingiae sp. nov., Colwellia hornerae sp. nov., Colwellia rossensis sp. nov. and Colwellia psychrotropica sp. nov.: psychrophilic Antarctic species with the ability to synthesize docosahexaenoic acid (22 : 6 omega 3). *International Journal of Systematic Bacteriology* 48**,** 1171-1180.

Bowman, J.P., Mccammon, S.A., Brown, J.L., and Mcmeekin, T.A. (1998b). Glaciecola punicea gen. nov., sp. nov. and Glaciecola pallidula gen. nov., sp. nov.: psychrophilic bacteria from Antarctic sea-ice habitats. *International Journal of Systematic Bacteriology* 48**,** 1213-1222.

Bowman, J.P., Mccammon, S.A., Brown, J.L., Nichols, P.D., and Mcmeekin, T.A. (1997a). Psychroserpens burtonensis gen. nov, sp. nov, and Gelidibacter algens gen. nov, sp. nov, psychrophilic bacteria isolated from Antarctic lacustrine and sea ice habitats. *International Journal of Systematic Bacteriology* 47**,** 670-677.

Bowman, J.P., Mccammon, S.A., Lewis, T., Skerratt, J.H., Brown, J.L., Nichols, D.S., and Mcmeekin, T.A. (1998c). *Psychroflexus torquis* gen. nov., sp. nov. a psychrophilic species from Antarctic sea ice, and reclassification of *Flavobacterium gondwanense* (Dobson et al. 1993) as *Psychroflexus gondwanense* gen. nov., comb. nov. *Microbiology* 144**,** 1601-1609.

Bowman, J.P., Mccammon, S.A., Nichols, D.S., Skerratt, J.H., Rea, S.M., Nichols, P.D., and Mcmeekin, T.A. (1997b). Shewanella gelidimarina sp. nov. and Shewanella frigidimarina sp. nov., novel Antarctic species with the ability to produce eicosapentaenoic acid (20:5 omega 3) and grow anaerobically by dissimilatory Fe(III) reduction. *International Journal of Systematic Bacteriology* 47**,** 1040-1047.

Bowman, J.P., Mccammon, S.A., and Skerrat, J.H. (1997c). *Methylosphaera hansonii* gen. nov., sp. nov., a psychrophilic, group I methanotroph from Antarctic marine-salinity, meromictic lakes. *Microbiology* 143**,** 1451-1459.

Bowman, J.P., and Nichols, D.S. (2005). Novel members of the family Flavobacteriaceae from Antarctic maritime habitats including Subsaximicrobium wynnwilliamsii gen. nov., sp nov., Subsaximicrobium saxinquililnus sp nov., Subsaxibacter broadyi gen. nov., sp nov., Lacinutrix copepodicola gen. nov., sp nov., and novel species of the genera Bizionia, Gelidibacter and Gillisia. *International Journal of Systematic and Evolutionary Microbiology* 55**,** 1471-1486.

Bowman, J.P., Nichols, D.S., and Mcmeekin, T.A. (1997d). Psychrobacter glacincola sp. nov, a halotolerant, psychrophilic bacterium isolated from Antarctic sea ice. *Systematic and Applied Microbiology* 20**,** 209-215.

Bozal, N., Montes, M.J., and Mercade, E. (2007). Pseudomonas guineae sp nov., a novel psychrotolerant bacterium from an Antarctic environment. *International Journal of Systematic and Evolutionary Microbiology* 57**,** 2609-2612.

Bozal, N., Montes, M.J., Tudela, E., and Guinea, J. (2003). Characterization of several Psychrobacter strains isolated from Antarctic environments and description of Psychrobacter luti sp nov and Psychrobacter fozii sp nov. *International Journal of Systematic and Evolutionary Microbiology* 53**,** 1093-1100.

Bozal, N., Montes, M.J., Tudela, E., Jimenez, F., and Guinea, J. (2002). Shewanella frigidimarina and Shewanella livingstonensis sp nov isolated from Antarctic coastal areas. *International Journal of Systematic and Evolutionary Microbiology* 52**,** 195-205.

Bozal, N., Tudela, E., Rossellomora, R., Lalucat, J., and Guinea, J. (1997). Pseudoalteromonas antarctica sp nov, isolated from an Antarctic coastal environment. *International Journal of Systematic Bacteriology* 47**,** 345-351.

Busse, H.J., Denner, E.B.M., Buczolits, S., Salkinoja-Salonen, M., Bennasar, A., and Kampfer, P. (2003). Sphingomonas aurantiaca sp nov., Sphingomonas aerolata sp nov and Sphingomonas faeni sp nov., air- and dustborne and Antarctic, orange-pigmented, psychrotolerant bacteria, and emended description of the genus Sphingomonas. *International Journal of Systematic and Evolutionary Microbiology* 53**,** 1253-1260.

Carrion, O., Minana-Galbis, D., Montes, M.J., and Mercade, E. (2011). Pseudomonas deceptionensis sp nov., a psychrotolerant bacterium from the Antarctic. *International Journal of Systematic and Evolutionary Microbiology* 61**,** 2401-2405.

Chaturvedi, P., Prabahar, V., Manorama, R., Pindi, P.K., Bhadra, B., Begum, Z., and Shivaji, S. (2008). Exiguobacterium soli sp nov., a psychrophilic bacterium from the McMurdo Dry Valleys, Antarctica. *International Journal of Systematic and Evolutionary Microbiology* 58**,** 2447-2453.

Chen, C.X., Zhang, X.Y., Liu, C., Yu, Y., Liu, A., Li, G.W., Li, H., Chen, X.L., Chen, B., Zhou, B.C., and Zhang, Y.Z. (2013). Pseudorhodobacter antarcticus sp nov., isolated from Antarctic intertidal sandy sediment, and emended description of the genus Pseudorhodobacter Uchino et al. 2002 emend. Jung et al. 2012. *International Journal of Systematic and Evolutionary Microbiology* 63**,** 849-854.

Chen, M., Xiao, X., Wang, P., Zeng, X., and Wang, F.P. (2005). Arthrobacter ardleyensis sp nov., isolated from Antarctic lake sediment and deep-sea sediment. *Archives of Microbiology* 183**,** 301-305.

Choi, J.Y., Kim, J.H., and Lee, P.C. (2018). Flavobacterium kingsejongi sp nov., a carotenoid-producing species isolated from Antarctic penguin faeces. *International Journal of Systematic and Evolutionary Microbiology* 68**,** 911-916.

Choi, S.G., Jeon, S.H., Lee, J.B., Joo, E.S., Lim, S., Jung, H.Y., and Kim, M.K. (2016). Deinococcus rubellus sp nov., bacteria isolated from the muscle of antarctic fish. *Journal of Microbiology* 54**,** 796-801.

Choi, T.H., Lee, H.K., Lee, K., and Cho, J.C. (2007). Ulvibacter antarcticus sp nov., isolated from Antarctic coastal seawater. *International Journal of Systematic and Evolutionary Microbiology* 57**,** 2922-2925.

Chua, M.J., Campen, R.L., Wahl, L., Grzymski, J.J., and Mikucki, J.A. (2018). Genomic and physiological characterization and description of Marinobacter gelidimuriae sp nov., a psychrophilic, moderate halophile from Blood Falls, an antarctic subglacial brine. *Fems Microbiology Ecology* 94.

Collins, M.D., Lawson, P.A., Labrenz, M., Tindall, B.J., Weiss, N., and Hirsch, P. (2002). Nesterenkonia lacusekhoensis sp nov., isolated from hypersaline Ekho Lake, East Antarctica, and emended description of the genus Nesterenkonia. *International Journal of Systematic and Evolutionary Microbiology* 52**,** 1145-1150.

Da, X.Y., Jiang, F., Chang, X.L., Ren, L.Z., Qiu, X., Kan, W.J., Zhang, Y.M., Deng, S.S., Fang, C.X., and Peng, F. (2015). Pedobacter ardleyensis sp nov., isolated from soil in Antarctica. *International Journal of Systematic and Evolutionary Microbiology* 65**,** 3841-3846.

Debruyne, L., Broman, T., Bergstrom, S., Olsen, B., On, S.L.W., and Vandamme, P. (2010). Campylobacter subantarcticus sp nov., isolated from birds in the sub-Antarctic region. *International Journal of Systematic and Evolutionary Microbiology* 60**,** 815-819.

Deng, S.S., Jiang, F., Chang, X.L., Qu, Z.H., Ren, L.Z., Zhang, Y.M., Kan, W.J., Da, X.Y., Qiu, X., Kim, M., Fang, C.X., and Peng, F. (2015). Roseovarius antarcticus sp nov., isolated from a decayed whale bone. *International Journal of Systematic and Evolutionary Microbiology* 65**,** 2326-2333.

Denner, E.B.M., Mark, B., Busse, H.J., Turkiewicz, M., and Lubitz, W. (2001). Psychrobacter proteolyticus sp nov., a psychrotrophic, halotolerant bacterium isolated from the antarctic krill Euphausia superba Dana, excreting a cold-adapted metalloprotease. *Systematic and Applied Microbiology* 24**,** 44-53.

Dobson, S.J., Colwell, R.R., Mcmeekin, T.A., and Franzmann, P.D. (1993). DIRECT SEQUENCING OF THE POLYMERASE CHAIN REACTION-AMPLIFIED 16S RIBOSOMAL-RNA GENE OF FLAVOBACTERIUM-GONDWANENSE SP-NOV AND FLAVOBACTERIUM-SALEGENS SP-NOV, 2 NEW SPECIES FROM A HYPERSALINE ANTARCTIC LAKE. *International Journal of Systematic Bacteriology* 43**,** 77-83.

Dong, N., Li, H.R., Yuan, M., Zhang, X.H., and Yu, Y. (2015). Deinococcus antarcticus sp nov., isolated from soil. *International Journal of Systematic and Evolutionary Microbiology* 65**,** 331-335.

Finore, I., Orlando, P., Di Donato, P., Leone, L., Nicolaus, B., and Poli, A. (2016). Nesterenkonia aurantiaca sp nov., an alkaliphilic actinobacterium isolated from Antarctica. *International Journal of Systematic and Evolutionary Microbiology* 66**,** 1554-1560.

Franzmann, P.D., Hopfl, P., Weiss, N., and Tindall, B.J. (1991). PSYCHOTROPHIC, LACTIC ACID-PRODUCING BACTERIA FROM ANOXIC WATERS IN ACE LAKE, ANTARCTICA - CARNOBACTERIUM-FUNDITUM SP-NOV AND CARNOBACTERIUM-ALTERFUNDITUM SP-NOV. *Archives of Microbiology* 156**,** 255-262.

Franzmann, P.D., Liu, Y.T., Balkwill, D.L., Aldrich, H.C., Demacario, E.C., and Boone, D.R. (1997). Methanogenium frigidum sp. nov., a psychrophilic, H-2-using methanogen from Ace Lake, Antarctica. *International Journal of Systematic Bacteriology* 47**,** 1068-1072.

Franzmann, P.D., Springer, N., Ludwig, W., Demacario, E.C., and Rohde, M. (1992). A methanogenic archaeon from Ace Lake, Antarctica - Methanococcoides burtonii sp nov. *Systematic and Applied Microbiology* 15**,** 573-581.

Franzmann, P.D., Stackebrandt, E., Sanderson, K., Volkman, J.K., Cameron, D.E., Stevenson, P.L., Mcmeekin, T.A., and Burton, H.R. (1988). Halobacterium lacusprofundi sp. nov., a Halophilic Bacterium Isolated from Deep Lake, Antarctica. *Systematic and Applied Microbiology* 11**,** 20-27.

Fruhling, A., Schumann, P., Hippe, H., Straubler, B., and Stackebrandt, E. (2002). Exiguobacterium undae sp nov and Exiguobacterium antarcticum sp nov. *International Journal of Systematic and Evolutionary Microbiology* 52**,** 1171-1176.

Fukuda, W., Chino, Y., Araki, S., Kondo, Y., Imanaka, H., Kanai, T., Atomi, H., and Imanaka, T. (2014). Polymorphobacter multimanifer gen. nov., sp nova, a polymorphic bacterium isolated from antarctic white rock. *International Journal of Systematic and Evolutionary Microbiology* 64**,** 2034-2040.

Fukuda, W., Kimura, T., Araki, S., Miyoshi, Y., Atomi, H., and Imanaka, T. (2013). Lysobacter oligotrophicus sp nov., isolated from an Antarctic freshwater lake in Antarctica. *International Journal of Systematic and Evolutionary Microbiology* 63**,** 3313-3318.

Fukuda, W., Yamada, K., Miyoshi, Y., Okuno, H., Atomi, H., and Imanaka, T. (2012). Rhodoligotrophos appendicifer gen. nov., sp nov., an appendaged bacterium isolated from a freshwater Antarctic lake. *International Journal of Systematic and Evolutionary Microbiology* 62**,** 1945-1950.

Ganzert, L., Bajerski, F., Mangelsdorf, K., Lipski, A., and Wagner, D. (2011a). Arthrobacter livingstonensis sp. nov. and Arthrobacter cryotolerans sp. nov., salt-tolerant and psychrotolerant species from Antarctic soil. *International Journal of Systematic and Evolutionary Microbiology* 61**,** 979-984.

Ganzert, L., Bajerski, F., Mangelsdorf, K., Lipski, A., and Wagner, D. (2011b). Leifsonia psychrotolerans sp nov., a psychrotolerant species of the family Microbacteriaceae from Livingston Island, Antarctica. *International Journal of Systematic and Evolutionary Microbiology* 61**,** 1938-1943.

Gosink, J.J., Herwig, R.P., and Staley, J.T. (1997). Octadecabacter arcticus gen. nov., sp. nov., and O-antarcticus, sp. nov., nonpigmented, psychrophilic gas vacuolate bacteria from polar sea ice and water (vol 20, pg 356, 1997). *Systematic and Applied Microbiology* 20**,** 512-512.

Gupta, P., Reddy, G.S.N., Delille, D., and Shivaji, S. (2004). Arthrobacter gangotriensis sp nov and Arthrobacter kerguelensis sp nov from Antarctica. *International Journal of Systematic and Evolutionary Microbiology* 54**,** 2375-2378.

Han, Y.Q., Zhao, R., Yu, T., Li, Z., and Zhang, X.H. (2016). Allohahella marinimesophila gen. nov., sp nov., isolated from seawater and reclassification of Hahella antarctica as Allohahella antarctica comb. nov. *International Journal of Systematic and Evolutionary Microbiology* 66**,** 3207-3213.

Heuchert, A., Glockner, F.O., Amann, R., and Fischer, U. (2004). Psychrobacter nivimaris sp nov, a heterotrophic bacterium attached to organic particles isolated from the South Atlantic (Antarctica). *Systematic and Applied Microbiology* 27**,** 399-406.

Hirsch, P., Gallikowski, C.A., Siebert, J., Peissl, K., Kroppenstedt, R., Schumann, P., Stackebrandt, E., and Anderson, R. (2004a). Deinococcus frigens sp nov., Deinococcus saxicola sp nov., and Deinococcus marmoris sp nov., low temperature and draught-tolerating, UV-resistant bacteria from continental Antarctica. *Systematic and Applied Microbiology* 27**,** 636-645.

Hirsch, P., Ludwig, W., Hethke, C., Sittig, M., Hoffmann, B., and Gallikowski, C.A. (1998). *Hymenobacter roseosalivarius* gen. nov., sp. nov. from continental Antarctic soils and sandstone: Bacteria of the Cytophaga/Flavobacterium/Bacteroides line of phylogenetic descent. *Systematic and Applied Microbiology* 21**,** 374-383.

Hirsch, P., Mevs, U., Kroppenstedt, R.M., Schumann, P., and Stackebrandt, E. (2004b). Cryptoenclolithic actinomycetes from antarctic sandstone rock samples: Micromonospora endolithica sp nov and two isolates related to micromonospora coerulea Jensen 1932. *Systematic and Applied Microbiology* 27**,** 166-174.

Hong, S.G., Lee, Y.K., Yim, J.H., Chun, J., and Lee, H.K. (2008). Sanguibacter antarcticus sp nov., isolated from Antarctic sea sand. *International Journal of Systematic and Evolutionary Microbiology* 58**,** 50-52.

Huang, Y., Wei, Z.Y., Danzeng, W., Kim, M.C., Zhu, G.X., Zhang, Y.M., Liu, Z.B., and Peng, F. (2017). Sphingomonas antarctica sp nov., isolated from Antarctic tundra soil. *International Journal of Systematic and Evolutionary Microbiology* 67**,** 4064-4068.

Humphry, D.R., George, A., Black, G.W., and Cummings, S.P. (2001). Flavobacterium frigidarium sp nov., an aerobic, psychrophilic, xylanolytic and laminarinolytic bacterium from Antarctica. *International Journal of Systematic and Evolutionary Microbiology* 51**,** 1235-1243.

Huo, Y.Y., Li, Z.Y., You, H., Wang, C.S., Post, A.F., Oren, A., and Xu, X.W. (2014). Oceanicola antarcticus sp nov and Oceanicola flagellatus sp nov., moderately halophilic bacteria isolated from seawater. *International Journal of Systematic and Evolutionary Microbiology* 64**,** 2975-2979.

Hwang, C.Y., Il Zhang, G., Kang, S.H., Kim, H.J., and Cho, B.C. (2009). Pseudomonas pelagia sp nov., isolated from a culture of the Antarctic green alga Pyramimonas gelidicola. *International Journal of Systematic and Evolutionary Microbiology* 59**,** 3019-3024.

Hwang, C.Y., Lee, I., Hwang, Y.J., Yoon, S.J., Lee, W.S., and Cho, B.C. (2016). Pseudoalteromonas neustonica sp. nov., isolated from the sea surface microlayer of the Ross Sea (Antarctica), and emended description of the genus Pseudoalteromonas. *International Journal of Systematic and Evolutionary Microbiology* 66**,** 3377-3382.

Imperio, T., Viti, C., and Marri, L. (2008). Alicyclobacillus pohliae sp nov., a thermophilic, endospore-forming bacterium isolated from geothermal soil of the north-west slope of Mount Melbourne (Antarctica). *International Journal of Systematic and Evolutionary Microbiology* 58**,** 221-225.

Irgens, R.L., Gosink, J.J., and Staley, J.T. (1996). Polaromonas vacuolata gen nov, sp nov, a psychrophilic, marine, gas vacuolate bacterium from Antarctica. *International Journal of Systematic Bacteriology* 46**,** 822-826.

Jiang, F., Danzeng, W.M., Zhang, Y.M., Zhang, Y., Jiang, L., Liu, J., Lu, L., Fan, W., and Peng, F. (2018). Hymenobacter rubripertinctus sp nov., isolated from Antarctic tundra soil. *International Journal of Systematic and Evolutionary Microbiology* 68**,** 663-668.

Junge, K., Gosink, J.J., Hoppe, H.G., and Staley, J.T. (1998). Arthrobacter, Brachybacterium and Planococcus isolates identified from Antarctic Sea ice brine. Description of Planococcus mcmeekinii, sp. nov. *Systematic and Applied Microbiology* 21**,** 306-314.

Kampfer, P., Irgang, R., Poblete-Morales, M., Glaeser, S.P., Cortez-San Martin, M., and Avendano-Herrera, R. (2017). Psychromonas aquatilis sp nov., isolated from seawater samples obtained in the Chilean Antarctica. *International Journal of Systematic and Evolutionary Microbiology* 67**,** 1306-1311.

Kim, B.C., Oh, H.W., Kim, H., Park, D.S., Hong, S.G., Lee, H.K., and Bae, K.S. (2013). Polaribacter sejongensis sp nov., isolated from Antarctic soil, and emended descriptions of the genus Polaribacter, Polaribacter butkevichii and Polaribacter irgensii. *International Journal of Systematic and Evolutionary Microbiology* 63**,** 4000-4005.

Kim, E.H., Jeong, H.J., Lee, Y.K., Moon, E.Y., Cho, J.C., Lee, H.K., and Hong, S.G. (2011). Actimicrobium antarcticum gen. nov., sp nov., of the Family Oxalobacteraceae, Isolated from Antarctic Coastal Seawater. *Current Microbiology* 63**,** 213-217.

Kim, J.H., Choi, B.H., Jo, M., Kim, S.C., and Lee, P.C. (2014). Flavobacterium faecale sp nov., an agarase-producing species isolated from stools of Antarctic penguins. *International Journal of Systematic and Evolutionary Microbiology* 64**,** 2884-2890.

Kim, J.H., Kang, H.J., Yu, B.J., Kim, S.C., and Lee, P.C. (2015). Planococcus faecalis sp nov., a carotenoid-producing species isolated from stools of Antarctic penguins. *International Journal of Systematic and Evolutionary Microbiology* 65**,** 3373-3378.

Kojima, H., Watanabe, M., Tokizawa, R., Shinohara, A., and Fukui, M. (2016). Hymenobacter nivis sp nov., isolated from red snow in Antarctica. *International Journal of Systematic and Evolutionary Microbiology* 66**,** 4821-4825.

Kosina, M., Bartak, M., Maslanova, I., Pascutti, A.V., Sedo, O., Lexa, M., and Sedlacek, I. (2013). Pseudomonas prosekii sp nov., a Novel Psychrotrophic Bacterium from Antarctica. *Current Microbiology* 67**,** 637-646.

Kosina, M., Svec, P., Cernohlavkova, J., Bartak, M., Snopkova, K., De Vos, P., and Sedlacek, I. (2016). Description of Pseudomonas gregormendelii sp nov., a Novel Psychrotrophic Bacterium from James Ross Island, Antarctica. *Current Microbiology* 73**,** 84-90.

Kralova, S., Svec, P., Busse, H.J., Stankova, E., Vaczi, P., and Sedlacek, I. (2018). Flavobacterium chryseum sp nov and Flavobacterium psychroterrae sp nov., novel environmental bacteria isolated from Antarctica. *International Journal of Systematic and Evolutionary Microbiology* 68**,** 3132-3139.

Kwon, Y.M., Yang, S.H., Kwon, K.K., and Kim, S.J. (2014). Nonlabens antarcticus sp nov., a psychrophilic bacterium isolated from glacier ice, and emended descriptions of Nonlabens marinus Park et al. 2012 and Nonlabens agnitus Yi and Chun 2012. *International Journal of Systematic and Evolutionary Microbiology* 64**,** 400-405.

Labrenz, M., Lawson, P.A., Tindal, B.J., Collins, M.D., and Hirsch, P. (2005). Roseisalinus antarcticus gen. nov., sp nov., a novel aerobic bacteriochlorophyll a-producing alpha-proteobacterium isolated from hypersaline Ekho Lake, Antarctica. *International Journal of Systematic and Evolutionary Microbiology* 55**,** 41-47.

Labrenz, M., Lawson, P.A., Tindall, B.J., Collins, M.D., and Hirsch, P. (2003). Saccharospirillum impatiens gen. nov., sp nov., a novel gamma-Proteobacterium isolated from hypersaline Ekho Lake (East Antarctica). *International Journal of Systematic and Evolutionary Microbiology* 53**,** 653-660.

Labrenz, M., Tindall, B.J., Lawson, P.A., Collins, M.D., Schumann, P., and Hirsch, P. (2000). Staleya guttiformis gen. nov., sp nov and Sulfitobacter brevis sp nov., alpha-3-Proteobacteria from hypersaline, heliothermal and meromictic antarctic Ekho Lake. *International Journal of Systematic and Evolutionary Microbiology* 50**,** 303-313.

Lai, Q.L., Li, G.Z., Liu, X.P., Du, Y.P., Sun, F.Q., and Shao, Z.Z. (2015). Pseudooceanicola atlanticus gen. nov sp nov., isolated from surface seawater of the Atlantic Ocean and reclassification of Oceanicola batsensis, Oceanicola marinus, Oceanicola nitratireducens, Oceanicola nanhaiensis, Oceanicola antarcticus and Oceanicola flagellatus, as Pseudooceanicola batsensis comb. nov., Pseudooceanicola marinus comb. nov., Pseudooceanicola nitratireducens comb. nov., Pseudooceanicola nanhaiensis comb. nov., Pseudooceanicola antarcticus comb. nov., and Pseudooceanicola flagellatus comb. nov. *Antonie Van Leeuwenhoek International Journal of General and Molecular Microbiology* 107**,** 1065-1074.

Lawson, P.A., Collins, M.D., Schumann, P., Tindall, B.J., Hirsch, P., and Labrenz, M. (2000). New LL-diaminopimelic acid-containing actinomycetes from hypersaline, heliothermal and meromictic Antarctic Ekho Lake: Nocardioides aquaticus sp nov and Friedmannielly lacustris sp nov. *Systematic and Applied Microbiology* 23**,** 219-229.

Le Roes-Hill, M., Rohland, J., Meyers, P.R., Cowan, D.A., and Burton, S.G. (2009). Streptomyces hypolithicus sp. nov., isolated from an Antarctic hypolith community. *International Journal of Systematic and Evolutionary Microbiology* 59**,** 2032-2035.

Lee, Kiyoung, Lee, H.K., Choi, T.H., Kim, K.M.T., and Cho, J.C. (2007a). Granulosicoccaceae fam nov, to include Granulosicoccus antarcticus gen nov, sp nov, a non-phototrophic, obligately aerobic chemoheterotroph in the order Chromatiales, isolated from antarctic Seawater. *Journal of Microbiology and Biotechnology* 17**,** 1483-1490.

Lee, J.B., Jeon, S.H., Choi, S.G., Jung, H.Y., Kim, M.K., and Srinivasan, S. (2016). Bacillus piscis sp nov., a novel bacterium isolated from the muscle of the antarctic fish Dissostichus mawsoni. *Journal of Microbiology* 54**,** 809-813.

Lee, K., Lee, H.K., and Cho, J.C. (2008). Hahella antarctica sp nov., isolated from Antarctic seawater. *International Journal of Systematic and Evolutionary Microbiology* 58**,** 353-356.

Lee, K., Lee, H.K., Choi, T.H., and Cho, J.C. (2007b). Robiginftomaculum antarcticum gen. nov., sp nov., a member of the family Hyphomonadaceae, from Antarctic seawater. *International Journal of Systematic and Evolutionary Microbiology* 57**,** 2595-2599.

Lee, K., Lee, H.K., Choi, T.H., and Cho, J.C. (2007c). Sejongia marina sp nov., isolated from Antarctic seawater. *International Journal of Systematic and Evolutionary Microbiology* 57**,** 2917-2921.

Lee, Y.M., Hwang, C.Y., Lee, I., Jung, Y.J., Cho, Y., Baek, K., Hong, S.G., Kim, J.H., Chun, J., and Lee, H.K. (2014). Lacinutrix jangbogonensis sp nov., a psychrophilic bacterium isolated from Antarctic marine sediment and emended description of the genus Lacinutrix. *Antonie Van Leeuwenhoek International Journal of General and Molecular Microbiology* 106**,** 527-533.

Li, A.Z., Han, X.B., Lin, L.Z., Zhang, M.X., and Zhu, H.H. (2018a). Gramella antarctica sp nov., isolated from marine surface sediment. *International Journal of Systematic and Evolutionary Microbiology* 68**,** 358-363.

Li, A.Z., Lin, L.Z., Zhang, M.X., and Zhu, H.H. (2017). Arenibacter antarcticus sp nov., isolated from marine sediment. *International Journal of Systematic and Evolutionary Microbiology* 67**,** 4601-4605.

Li, A.Z., Lin, L.Z., Zhang, M.X., and Zhu, H.H. (2018b). Antarcticibacterium flavum gen. nov., sp nov., isolated from marine sediment. *International Journal of Systematic and Evolutionary Microbiology* 68**,** 254-259.

Li, H.J., Zhang, X.Y., Chen, C.X., Zhang, Y.J., Gao, Z.M., Yu, Y., Chen, X.L., Chen, B., and Zhang, Y.Z. (2011a). Zhongshania antarctica gen. nov., sp nov and Zhongshania guokunii sp nov., gammaproteobacteria respectively isolated from coastal attached (fast) ice and surface seawater of the Antarctic. *International Journal of Systematic and Evolutionary Microbiology* 61**,** 2052-2057.

Li, H.R., Yu, Y., Luo, W., and Zeng, Y.X. (2010). Marisediminicola antarctica gen. nov., sp. nov., an actinobacterium isolated from the Antarctic. *International Journal of Systematic and Evolutionary Microbiology* 60**,** 2535-2539.

Li, J., Tian, X.-P., Zhu, T.-J., Yang, L.-L., and Li, W.-J. (2011b). Streptomyces fildesensis sp. nov., a novel streptomycete isolated from Antarctic soil. *Antonie van Leeuwenhoek* 100**,** 537.

Liu, C., Chen, C.X., Zhang, X.Y., Yu, Y., Liu, A., Li, G.W., Chen, X.L., Chen, B., Zhou, B.C., and Zhang, Y.Z. (2012). Marinobacter antarcticus sp nov., a halotolerant bacterium isolated from Antarctic intertidal sandy sediment. *International Journal of Systematic and Evolutionary Microbiology* 62**,** 1838-1844.

Liu, C., Zhang, X.Y., Su, H.N., Zhou, M.Y., Chen, B., Li, H., Chen, X.L., Zhao, D.L., Zhou, B.C., Shi, M., and Zhang, Y.Z. (2014). Puniceibacterium antarcticum gen. nov., sp nov., isolated from seawater. *International Journal of Systematic and Evolutionary Microbiology* 64**,** 1566-1572.

Liu, H.C., Xu, Y., Ma, Y.H., and Zhou, P.J. (2000). Characterization of Micrococcus antarcticus sp nov., a psychrophilic bacterium from Antarctica. *International Journal of Systematic and Evolutionary Microbiology* 50**,** 715-719.

Liu, J.J., Zhang, X.Q., Pan, J., Sun, C., Zhang, Y., Li, C.Q., Zhu, X.F., and Wu, M. (2013). Aequorivita viscosa sp nov., isolated from an intertidal zone, and emended descriptions of Aequorivita antarctica and Aequorivita capsosiphonis. *International Journal of Systematic and Evolutionary Microbiology* 63**,** 3192-3196.

Logan, N.A., De Clerck, E., Lebbe, L., Verhelst, A., Goris, J., Forsyth, G., Rodriguez-Diaz, M., Heyndrickx, M., and De Vos, P. (2004). Paenibacillus cineris sp nov and Paenibacillus cookii sp nov., from Antarctic volcanic soils and a gelatin-processing plant. *International Journal of Systematic and Evolutionary Microbiology* 54**,** 1071-1076.

Logan, N.A., Lebbe, L., Hoste, B., Goris, J., Forsyth, G., Heyndrickx, M., Murray, B.L., Syme, N., Wynn-Williams, D.D., and De Vos, P. (2000). Aerobic endospore-forming bacteria from geothermal environments in northern Victoria Land, Antarctica, and Candlemas Island, South Sandwich archipelago, with the proposal of Bacillus fumarioli sp. nov. *International Journal of Systematic and Evolutionary Microbiology* 50**,** 1741-1753.

Lopez, N.I., Pettinari, M.J., Stackebrandt, E., Tribelli, P.M., Potter, M., Steinbuchel, A., and Mendez, B.S. (2009). Pseudomonas extremaustralis sp nov., a Poly(3-hydroxybutyrate) Producer Isolated from an Antarctic Environment. *Current Microbiology* 59**,** 514-519.

Lozal, N., Montes, M.J., Minana-Galbis, D., Manresa, A., and Mercade, E. (2009). Shewanella vesiculosa sp nov., a psychrotolerant bacterium isolated from an Antarctic coastal area. *International Journal of Systematic and Evolutionary Microbiology* 59**,** 336-340.

Madigan, M.T., Jung, D.O., Woese, C.R., and Achenbach, L.A. (2000). *Rhodoferax antarcticus* sp. nov., a moderately psychrophilic purple nonsulfur bacterium isolated from an Antarctic microbial mat. *Archives of Microbiology* 173**,** 269-277.

Mccammon, S.A., and Bowman, J.P. (2000). Taxonomy of Antarctic Flavobacterium species: description of Flavobacterium gillisiae sp nov., Flavobacterium tegetincola sp nov and Flavobacterium xanthum sp nov., nom. rev. and reclassification of Flavobacterium salegens as Salegentibacter salegens gen. nov., comb. nov. *International Journal of Systematic and Evolutionary Microbiology* 50**,** 1055-1063.

Mccammon, S.A., Innes, B.H., Bowman, J.P., Franzmann, P.D., Dobson, S.J., Holloway, P.E., Skerratt, J.H., Nichols, P.D., and Rankin, L.M. (1998). Flavobacterium hibernum sp. nov., a lactose-utilizing bacterium from a freshwater Antarctic lake. *International Journal of Systematic Bacteriology* 48**,** 1405-1412.

Mcguire, A.J., Franzmann, P.D., and Mcmeekin, T.A. (1987). FLECTOBACILLUS-GLOMERATUS SP-NOV, A CURVED, NONMOTILE, PIGMENTED BACTERIUM ISOLATED FROM ANTARCTIC MARINE ENVIRONMENTS. *Systematic and Applied Microbiology* 9**,** 265-272.

Mevs, U., Stackebrandt, E., Schumann, P., Gallikowski, C.A., and Hirsch, P. (2000). *Modestobacter multiseptatus* gen. nov., sp nov., a budding actinomycete from soils of the Asgard Range (Transantarctic Mountains). *International Journal of Systematic and Evolutionary Microbiology* 50**,** 337-346.

Montes, M.J., Bozal, N., and Mercade, E. (2008). Marinobacter guineae sp nov., a novel moderately halophilic bacterium from an Antarctic environment. *International Journal of Systematic and Evolutionary Microbiology* 58**,** 1346-1349.

Montes, M.J., Mercade, E., Bozal, N., and Guinea, J. (2004). Paenibacillus antarcticus sp nov., a novel psychrotolerant organism from the Antarctic environment. *International Journal of Systematic and Evolutionary Microbiology* 54**,** 1521-1526.

Mou, Y.-Z., Qiu, X.-X., Zhao, M.-L., Cui, H.-L., Oh, D., and Dyall-Smith, M.L. (2012). Halohasta litorea gen. nov. sp. nov., and Halohasta litchfieldiae sp. nov., isolated from the Daliang aquaculture farm, China and from Deep Lake, Antarctica, respectively. *Extremophiles* 16**,** 895-901.

Mountfort, D.O., Rainey, F.A., Burghardt, J., Kaspar, H.F., and Stackebrandt, E. (1997). Clostridium vincentii sp nov, a new obligately anaerobic, saccharolytic, psychrophilic bacterium isolated from low-salinity pond sediment of the McMurdo Ice Shelf, Antarctica. *Archives of Microbiology* 167**,** 54-60.

Mountfort, D.O., Rainey, F.A., Burghardt, J., Kaspar, H.F., and Stackebrandt, E. (1998). Psychromonas antarcticus gen. nov., sp. nov., a new aerotolerant anaerobic, halophilic psychrophile isolated from pond sediment of the McMurdo Ice Shelf, Antarctica. *Archives of Microbiology* 169**,** 231-238.

Naqvi, S., Qin, Y.A., Tahir, A., and Stougaard, P. (2017). Pararhizobium antarcticum sp nov., isolated from Antarctic water samples. *International Journal of Systematic and Evolutionary Microbiology* 67**,** 1650-1655.

Nicolaus, B., Lama, L., Esposito, E., Manca, M.C., Diprisco, G., and Gambacorta, A. (1996). ''Bacillus thermoantarcticus'' sp nov, from Mount Melbourne, Antarctica: A novel thermophilic species. *Polar Biology* 16**,** 101-104.

Nogi, Y., Soda, K., and Oikawa, T. (2005). Flavobacterium frigidimaris sp nov., isolated from Antarctic seawater. *Systematic and Applied Microbiology* 28**,** 310-315.

Oh, H.M., Lee, K., and Cho, J.C. (2009). Lewinella antarctica sp nov., a marine bacterium isolated from Antarctic seawater. *International Journal of Systematic and Evolutionary Microbiology* 59**,** 65-68.

Pantucek, R., Sedlacek, I., Indrakova, A., Vrbovska, V., Maslanova, I., Kovarovic, V., Svec, P., Kralova, S., Kristofova, L., Keklakova, J., Petras, P., and Doskar, J. (2018). Staphylococcus edaphicus sp nov., Isolated in Antarctica, Harbors the mecC Gene and Genomic Islands with a Suspected Role in Adaptation to Extreme Environments. *Applied and Environmental Microbiology* 84.

Pikuta, E.V., Lyu, Z., Hoover, R.B., Liu, Y.C., Patel, N.B., Busse, H.J., and Lawson, P.A. (2017a). Williamwhitmania taraxaci gen. nov., sp nov., a proteolytic anaerobe with a novel type of cytology from Lake Untersee in Antarctica, description of Williamwhitmaniaceae fam. nov., and emendation of the order Bacteroidales Krieg 2012. *International Journal of Systematic and Evolutionary Microbiology* 67**,** 4132-4145.

Pikuta, E.V., Lyu, Z., Williams, M.D., Patel, N.B., Liu, Y.C., Hoover, R.B., Busse, H.J., Lawson, P.A., and Whitman, W.B. (2017b). Sanguibacter gelidistatuariae sp nov., a novel psychrotolerant anaerobe from an ice sculpture in Antarctica, and emendation of descriptions of the family Sanguibacteraceae, the genus Sanguibacter and species S. antarcticus, S. inulinus, S. kedieii, S. marinus, S. soli and S. suarezii. *International Journal of Systematic and Evolutionary Microbiology* 67**,** 1442-1450.

Pikuta, E.V., Menes, R.J., Bruce, A.M., Lyu, Z., Patel, N.B., Liu, Y., Hoover, R.B., Busse, H.J., Lawson, P.A., and Whitman, W.B. (2016). Raineyella antarctica gen. nov., sp nov., a psychrotolerant, D-amino-acid-utilizing anaerobe isolated from two geographic locations of the Southern Hemisphere. *International Journal of Systematic and Evolutionary Microbiology* 66**,** 5529-5536.

Pindi, P.K., Kishore, K.H., Reddy, G.S.N., and Shivaji, S. (2009). Description of Leifsonia kafniensis sp nov and Leifsonia antarctica sp nov. *International Journal of Systematic and Evolutionary Microbiology* 59**,** 1348-1352.

Pindi, P.K., Manorama, R., Begum, Z., and Shivaji, S. (2010). Arthrobacter antarcticus sp. nov., isolated from an Antarctic marine sediment. *International Journal of Systematic and Evolutionary Microbiology* 60**,** 2263-2266.

Poli, A., Esposito, E., Lama, L., Orlando, P., Nicolaus, G., De Appolonia, F., Gambacorta, A., and Nicolaus, B. (2006). Anoxybacillus amylolyticus sp nov., a thermophilic amylase producing bacterium isolated from Mount Rittmann (Antarctica). *Systematic and Applied Microbiology* 29**,** 300-307.

Poli, A., Esposito, E., Orlando, P., Lama, L., Giordano, A., De Appolonia, F., Nicolaus, B., and Gambacorta, A. (2007). Halomonas alkaliantarctica sp nov., isolated from saline lake Cape Russell in Antarctica, an alkalophilic moderately halophilic, exopolysaccharide-producing bacterium. *Systematic and Applied Microbiology* 30**,** 31-38.

Prabagaran, S.R., Suresh, K., Manorama, R., Delille, D., and Shivaji, S. (2005). Marinomonas ushuaiensis sp nov., isolated from coastal sea water in Ushuaia, Argentina, sub-Antarctica. *International Journal of Systematic and Evolutionary Microbiology* 55**,** 309-313.

Prabahar, V., Dube, S., Reddy, G.S.N., and Shivaji, S. (2004). Pseudonocardia antarctica sp nov an Actinomycetes from McMurdo Dry Valleys, Antarctica. *Systematic and Applied Microbiology* 27**,** 66-71.

Reddy, G.S.N., Aggarwal, R.K., Matsumoto, G.I., and Shivaji, S. (2000). Arthrobacter flavus sp, nov., a psychrophilic bacterium isolated from a pond in McMurdo Dry Valley, Antarctica. *International Journal of Systematic and Evolutionary Microbiology* 50**,** 1553-1561.

Reddy, G.S.N., Matsumoto, G.I., Schumann, P., Stackebrandt, E., and Shivaji, S. (2004). Psychrophilic pseudomonads from Antarctica: Pseudomonas antarctica sp nov., Pseudomonas meridiana sp nov and Pseudomonas proteolytica sp nov. *International Journal of Systematic and Evolutionary Microbiology* 54**,** 713-719.

Reddy, G.S.N., Matsumoto, G.I., and Shivaji, S. (2003a). Sporosarcina macmurdoensis sp nov., from a cyanobacterial mat sample from a pond in the McMurdo Dry Valleys, Antarctica. *International Journal of Systematic and Evolutionary Microbiology* 53**,** 1363-1367.

Reddy, G.S.N., Prakash, J.S.S., Matsumoto, G.I., Stackebrandt, E., and Shivaji, S. (2002). Arthrobacter roseus sp nov., a psychrophilic bacterium isolated from an Antarctic cyanobacterial mat sample. *International Journal of Systematic and Evolutionary Microbiology* 52**,** 1017-1021.

Reddy, G.S.N., Prakash, J.S.S., Prabahar, V., Matsumoto, G.I., Stackebrandt, E., and Shivaji, S. (2003b). Kocuria polaris sp nov., an orange-pigmented psychrophilic bacterium isolated from an Antarctic cyanobacterial mat sample. *International Journal of Systematic and Evolutionary Microbiology* 53**,** 183-187.

Reddy, G.S.N., Prakash, J.S.S., Srinivas, R., Matsumoto, G.I., and Shivaji, S. (2003c). Leifsonia rubra sp nov and Leifsonia aurea sp nov., psychrophiles from a pond in Antarctica. *International Journal of Systematic and Evolutionary Microbiology* 53**,** 977-984.

Reddy, G.S.N., Raghavan, P.U.M., Sarita, N.B., Prakash, J.S.S., Nagesh, N., Delille, D., and Shivaji, S. (2003d). Halomonas glaciei sp nov isolated from fast ice of Adelie Land, Antarctica. *Extremophiles* 7**,** 55-61.

Ren, Q.M., Yu, M., Li, Y.Y., Zhang, Y., Shi, X.C., Wu, Y.H., Su, Y., Wang, Y.A., Wang, X.L., and Zhang, X.H. (2018). Flavobacterium ovatum sp nov., a marine bacterium isolated from an Antarctic intertidal sandy beach. *International Journal of Systematic and Evolutionary Microbiology* 68**,** 795-800.

Rigonato, J., Gama, W.A., Alvarenga, D.O., Branco, L.H.Z., Brandini, F.P., Genuario, D.B., and Fiore, M.F. (2016). Aliterella atlantica gen. nov., sp nov., and Aliterella antarctica sp nov., novel members of coccoid Cyanobacteria. *International Journal of Systematic and Evolutionary Microbiology* 66**,** 2853-2861.

Rodriguez-Diaz, M., Lebbe, L., Rodelas, B., Heyrman, J., De Vos, P., and Logan, N.A. (2005). Paenibacillus wynnii sp nov., a novel species harbouring the nifH gene, isolated from Alexander Island, Antarctica. *International Journal of Systematic and Evolutionary Microbiology* 55**,** 2093-2099.

Sattley, W.M., Jung, D.O., and Madigan, M.T. (2008). Psychrosinus fermentans gen. nov., sp nov., a lactate-fermenting bacterium from near-freezing oxycline waters of a meromictic Antarctic lake. *Fems Microbiology Letters* 287**,** 121-127.

Sattley, W.M., and Madigan, M.T. (2010). Temperature and nutrient induced responses of Lake Fryxell sulfate-reducing prokaryotes and description of Desulfovibrio lacusfryxellense, sp nov., a pervasive, cold-active, sulfate-reducing bacterium from Lake Fryxell, Antarctica. *Extremophiles* 14**,** 357-366.

Schumann, P., Prauser, H., Rainey, F.A., Stackebrandt, E., and Hirsch, P. (1997). Friedmanniella antarctica gen nov, sp nov, an LL-diaminopimelic acid-containing actinomycete from antarctic sandstone. *International Journal of Systematic Bacteriology* 47**,** 278-283.

Sedlacek, I., Kralova, S., Kyrova, K., Maslanova, I., Busse, H.J., Stankova, E., Vrbovska, V., Nemec, M., Bartak, M., Holochova, P., Svec, P., and Pantucek, R. (2017a). Red-pink pigmented Hymenobacter coccineus sp nov., Hymenobacter lapidarius sp nov and Hymenobacter glacialis sp nov., isolated from rocks in Antarctica. *International Journal of Systematic and Evolutionary Microbiology* 67**,** 1975-1983.

Sedlacek, I., Pantucek, R., Kralova, S., Maslanova, I., Holochova, P., Stankova, E., Sobotka, R., Bartak, M., Busse, H.J., and Svec, P. (2017b). Mucilaginibacter terrae sp nov., isolated from Antarctic soil. *International Journal of Systematic and Evolutionary Microbiology* 67**,** 4002-4007.

See-Too, W.S., Salazar, S., Ee, R., Convey, P., Chan, K.G., and Peix, A. (2017). Pseudomonas versuta sp nov., isolated from Antarctic soil. *Systematic and Applied Microbiology* 40**,** 191-198.

Sheridan, P.P., Loveland-Curtze, J., Miteva, V.I., and Brenchley, J.E. (2003). Rhodoglobus vestalii gen. nov., sp nov., a novel psychrophilic organism isolated from an Antarctic Dry Valley lake. *International Journal of Systematic and Evolutionary Microbiology* 53**,** 985-994.

Shivaji, S., Ray, M.K., Rao, N.S., Saisree, L., Jagannadham, M.V., Kumar, G.S., Reddy, G.S.N., and Bhargava, P.M. (1992). SPHINGOBACTERIUM-ANTARCTICUS SP-NOV, A PSYCHROTROPHIC BACTERIUM FROM THE SOILS OF SCHIRMACHER OASIS, ANTARCTICA. *International Journal of Systematic Bacteriology* 42**,** 102-106.

Shivaji, S., Reddy, G.S., Sundareswaran, V.R., and Thomas, C. (2015). Description of Thalassospira lohafexi sp nov., isolated from Southern Ocean, Antarctica. *Archives of Microbiology* 197**,** 627-637.

Shivaji, S., Reddy, G.S.N., Raghavan, P.U.M., Sarita, N.B., and Delille, D. (2004). Psychrobacter salsus sp nov and Psychrobacter adeliensis sp nov isolated from fast ice from Adelie Land, Antarctica. *Systematic and Applied Microbiology* 27**,** 628-635.

Shivaji, S., Reddy, G.S.N., Suresh, K., Gupta, P., Chintalapati, S., Schumann, P., Stackebrandt, E., and Matsumoto, G.I. (2005). Psychrobacter vallis sp nov and Psychrobacter aquaticus sp nov., from Antarctica. *International Journal of Systematic and Evolutionary Microbiology* 55**,** 757-762.

Si, O.J., Yang, H.Y., Hwang, C.Y., Kim, S.J., Choi, S.B., Kim, J.G., Jung, M.Y., Kim, S.G., Roh, S.W., and Rhee, S.K. (2017). Kiloniella antarctica sp nov., isolated from a polynya of Amundsen Sea in Western Antarctic Sea. *International Journal of Systematic and Evolutionary Microbiology* 67**,** 2397-2402.

Silva, L.J., Souza, D.T., Genuario, D.B., Hoyos, H.a.V., Santos, S.N., Rosa, L.H., Zucchi, T.D., and Melo, I.S. (2018). Rhodococcus psychrotolerans sp nov., isolated from rhizosphere of Deschampsia antarctica. *Antonie Van Leeuwenhoek International Journal of General and Molecular Microbiology* 111**,** 629-636.

Snauwaert, I., Hoste, B., De Bruyne, K., Peeters, K., De Vuyst, L., Willems, A., and Vandamme, P. (2013). Carnobacterium iners sp nov., a psychrophilic, lactic acid-producing bacterium from the littoral zone of an Antarctic pond. *International Journal of Systematic and Evolutionary Microbiology* 63**,** 1370-1375.

Spring, S., Merkhoffer, B., Weiss, N., Kroppenstedt, R.M., Hippe, H., and Stackebrandt, E. (2003). Characterization of novel psychrophilic clostridia from an Antarctic microbial mat: description of Clostridium frigoris sp nov., Clostridium lacusfryxellense sp nov., Clostridium bowmanii sp nov and Clostridium psychrophilum sp nov and reclassification of Clostridium laramiense as Clostridium estertheticum subsp laramiense subsp nov. *International Journal of Systematic and Evolutionary Microbiology* 53**,** 1019-1029.

Srinivasan, S., Lim, S., Lim, J.H., Jung, H.Y., and Kim, M.K. (2017). Deinococcus rubrus sp nov., a Bacterium Isolated from Antarctic Coastal Sea Water. *Journal of Microbiology and Biotechnology* 27**,** 535-541.

Švec, P., Králová, S., Busse, H.-J., Kleinhagauer, T., Pantůček, R., Mašlaňová, I., Cnockaert, M., Vandamme, P., Staňková, E., Gelbíčová, T., Holochová, P., Barták, M., Kýrová, K., and Sedláček, I. (2017). Pedobacter jamesrossensis sp. nov., Pedobacter lithocola sp. nov., Pedobacter mendelii sp. nov. and Pedobacter petrophilus sp. nov., isolated from the Antarctic environment. *International Journal of Systematic and Evolutionary Microbiology* 67**,** 1499-1507.

Tahon, G., Tytgat, B., Lebbe, L., Carlier, A., and Willems, A. (2018). *Abditibacterium utsteinense* sp. nov., the first cultivated member of candidate phylum FBP, isolated from ice-free Antarctic soil samples. *Syst Appl Microbiol* 41**,** 279-290.

Van Trappen, S., Mergaert, J., and Swings, J. (2003). Flavobacterium gelidilacus sp nov., isolated from microbial mats in Antarctic lakes. *International Journal of Systematic and Evolutionary Microbiology* 53**,** 1241-1245.

Van Trappen, S., Mergaert, J., and Swings, J. (2004a). *Loktanella salsilacus* gen. nov., sp. nov., *Loktanella fryxellensis* sp. nov. and *Loktanella vestfoldensis* sp. nov., new members of the Rhodobacter group, isolated from microbial mats in Antarctic lakes. *International Journal of Systematic and Evolutionary Microbiology* 54**,** 1263-1269.

Van Trappen, S., Tan, T.L., Yang, J.F., Mergaert, J., and Swings, J. (2004b). Alteromonas stellipolaris sp nov., a novel, budding, prosthecate bacterium from Antarctic seas, and emended description of the genus Alteromonas. *International Journal of Systematic and Evolutionary Microbiology* 54**,** 1157-1163.

Van Trappen, S., Vandecandelaere, I., Mergaert, J., and Swings, J. (2004c). Flavobacterium degerlachei sp nov., Flavobacterium frigoris sp nov and Flavobacterium micromati sp nov., novel psychrophilic bacteria isolated from microbial mats in Antarctic lakes. *International Journal of Systematic and Evolutionary Microbiology* 54**,** 85-92.

Van Trappen, S., Vandecandelaere, I., Mergaert, J., and Swings, J. (2005). Flavobacterium fryxellicola sp nov and Flavobacterium psychrolimnae sp nov., novel psychrophilic bacteria isolated from microbial mats in Antarctic lakes. *International Journal of Systematic and Evolutionary Microbiology* 55**,** 769-772.

Van Trappen, S., Vandecandelaere, I., Mergaert, J.S., and Swings, J. (2004d). Gillisia limnaea gen. nov., sp nov., a new member of the family Flavobacteriaceae isolated from a microbial mat in Lake Fryxell, Antarctica. *International Journal of Systematic and Evolutionary Microbiology* 54**,** 445-448.

Wang, F.P., Gai, Y.B., Chen, M.X., and Xiao, X. (2009). Arthrobacter psychrochitiniphilus sp nov., a psychrotrophic bacterium isolated from Antarctica. *International Journal of Systematic and Evolutionary Microbiology* 59**,** 2759-2762.

Wang, J., Leiva, S., Huang, J., and Huang, Y. (2018). Amycolatopsis antarctica sp nov., isolated from the surface of an Antarctic brown macroalga. *International Journal of Systematic and Evolutionary Microbiology* 68**,** 2348-2356.

Wang, N., Xu, F., Zhang, X.Y., Chen, X.L., Qin, Q.L., Zhou, B.C., Zhang, Y.Z., and Shi, M. (2017). Changchengzhania lutea gen. nov., sp nov., a new member of the family Flavobacteriaceae isolated from Antarctic intertidal sediment. *International Journal of Systematic and Evolutionary Microbiology* 67**,** 5187-5192.

Wei, Z.Y., Huang, Y., Danzeng, W., Kim, M.C., Zhu, G.X., Zhang, Y.M., Liu, Z.B., and Peng, F. (2017). Flavitalea antarctica sp nov., isolated from Fildes Peninsula, Antarctica. *International Journal of Systematic and Evolutionary Microbiology* 67**,** 2258-2262.

Wu, Y.H., Yu, P.S., Zhou, Y.D., Xu, L., Wang, C.S., Wu, M., Oren, A., and Xu, X.W. (2013). Muricauda antarctica sp nov., a marine member of the Flavobacteriaceae isolated from Antarctic seawater. *International Journal of Systematic and Evolutionary Microbiology* 63**,** 3451-3456.

Xarnxidin, M., Wu, Y.H., Jian, S.L., Zhou, Y.D., Wang, C.S., Tohty, D., and Xu, X.W. (2016). Aquaticitalea lipolytica gen. nov., sp nov., isolated from Antarctic seawater. *International Journal of Systematic and Evolutionary Microbiology* 66**,** 2657-2663.

Yakimov, M.M., Giuliano, L., Gentile, G., Crisafi, E., Chernikova, T.N., Abraham, W.R., Lunsdorf, H., Timmis, K.N., and Golyshin, P.N. (2003). Oleispira antarctica gen. nov., sp nov., a novel hydrocarbonoclastic marine bacterium isolated from Antarctic coastal sea water. *International Journal of Systematic and Evolutionary Microbiology* 53**,** 779-785.

Yamada, K., Fukuda, W., Kondo, Y., Miyoshi, Y., Atomi, H., and Imanaka, T. (2011). Constrictibacter antarcticus gen. nov., sp nov., a cryptoendolithic micro-organism from Antarctic white rock. *International Journal of Systematic and Evolutionary Microbiology* 61**,** 1973-1980.

Yang, S.J., Oh, H.M., Chung, S., and Cho, J.C. (2009). Antarcticimonas flava gen. nov., sp nov., isolated from Antarctic coastal seawater. *Journal of Microbiology* 47**,** 517-523.

Yi, H., Oh, H.M., Lee, J.H., Kim, S.J., and Chun, J. (2005a). Flavobacterium antarcticum sp nov., a novel psychrotolerant bacterium isolated from the Antarctic. *International Journal of Systematic and Evolutionary Microbiology* 55**,** 637-641.

Yi, H., Yoon, H.I., and Chun, J. (2005b). Sejongia antarctica gen. nov., sp nov and Sejongia jeonii sp nov., isolated from the Antarctic. *International Journal of Systematic and Evolutionary Microbiology* 55**,** 409-416.

Yi, H.N., and Chun, J. (2006). Flavobacterium weaverense sp nov and Flavobacterium segetis sp nov., novel psychrophiles isolated from the Antarctic. *International Journal of Systematic and Evolutionary Microbiology* 56**,** 1239-1244.

Yu, Y., Li, H.R., Zeng, Y.X., Sun, K., and Chen, B. (2012). Pricia antarctica gen. nov., sp nov., a member of the family Flavobacteriaceae, isolated from Antarctic intertidal sediment. *International Journal of Systematic and Evolutionary Microbiology* 62**,** 2218-2223.

Yu, Y., Xin, Y.H., Liu, H.C., Chen, B., Sheng, J., Chi, Z.M., Zhou, P.J., and Zhang, D.C. (2008). Sporosarcina antarctica sp nov., a psychrophilic bacterium isolated from the Antarctic. *International Journal of Systematic and Evolutionary Microbiology* 58**,** 2114-2117.

Yu, Y., Yan, S.L., Li, H.R., and Zhang, X.H. (2011). Roseicitreum antarcticum gen. nov., sp nov., an aerobic bacteriochlorophyll a-containing alphaproteobacterium isolated from Antarctic sandy intertidal sediment. *International Journal of Systematic and Evolutionary Microbiology* 61**,** 2173-2179.

Zhang, G.I., Hwang, C.Y., Kang, S.H., and Cho, B.C. (2009). Maribacter antarcticus sp nov., a psychrophilic bacterium isolated from a culture of the Antarctic green alga Pyramimonas gelidicola. *International Journal of Systematic and Evolutionary Microbiology* 59**,** 1455-1459.

Zhang, H.Y., Hosoi-Tanabe, S., Nagata, S., Ban, S., and Imura, S. (2010a). Psychroflexus lacisalsi sp nov., a moderate halophilic bacterium isolated from a hypersaline lake (Hunazoko-Ike) in Antarctica. *Journal of Microbiology* 48**,** 160-164.

Zhang, X.Y., Zhang, Y.J., Yu, Y., Li, H.J., Gao, Z.M., Chen, X.L., Chen, B., and Zhang, Y.Z. (2010b). Neptunomonas antarctica sp nov., isolated from marine sediment. *International Journal of Systematic and Evolutionary Microbiology* 60**,** 1958-1961.

Zhang, Y.M., Jiang, F., Chang, X.L., Qiu, X., Ren, L.Z., Qu, Z.H., Deng, S.S., Da, X.Y., Fang, C.X., and Peng, F. (2016). Flavobacterium collinsense sp nov., isolated from a till sample of an Antarctic glacier. *International Journal of Systematic and Evolutionary Microbiology* 66**,** 172-177.

Zheng, R.C., Zhao, Y.M., Wang, L.Q., Chang, X.L., Zhang, Y.M., Da, X.Y., and Peng, F. (2016). Mucilaginibacter antarcticus sp nov., isolated from tundra soil. *International Journal of Systematic and Evolutionary Microbiology* 66**,** 5140-5144.

Zhou, M.Y., Zhang, X.Y., Yang, X.D., Zhang, Y.J., He, H.L., and Ning, D.L. (2017). Flavobacterium ardleyense sp nov., isolated from Antarctic soil. *International Journal of Systematic and Evolutionary Microbiology* 67**,** 3996-4001.

Zhou, M.Y., Zhang, Y.J., Zhang, X.Y., Yang, X.D., He, H.L., Ning, D.L., and Du, Z.J. (2018). Flavobacterium phocarum sp nov., isolated from soils of a seal habitat in Antarctica. *International Journal of Systematic and Evolutionary Microbiology* 68**,** 536-541.

Zhu, S.D., Lin, D.Q., Xiong, S.L., Wang, X., Xue, Z.H., Dong, B.X., Shen, X.D., Ma, X.D., Chen, J.G., and Yang, J.F. (2018). Carnobacterium antarcticum sp nov., a psychrotolerant, alkaliphilic bacterium isolated from sandy soil in Antarctica. *International Journal of Systematic and Evolutionary Microbiology* 68**,** 1672-1677.
